# Tumor-derived GLI1 promotes remodeling of the immune tumor microenvironment in melanoma

**DOI:** 10.1186/s13046-024-03138-0

**Published:** 2024-08-02

**Authors:** Alessandro Giammona, Chiara De Vellis, Enrica Crivaro, Luisa Maresca, Roberta Amoriello, Federica Ricci, Giulia Anichini, Silvia Pietrobono, David R. Pease, Martin E. Fernandez-Zapico, Clara Ballerini, Barbara Stecca

**Affiliations:** 1 Core Research Laboratory – Institute for Cancer Research and Prevention (ISPRO), Viale Pieraccini 6, 50139 Florence, Italy; 2 Department of Biotechnology, Chemistry and Pharmacy, University of Siena, Siena, Italy; 3 Department of Experimental and Clinical Medicine, University of Florence, Viale Pieraccini 6, 50139 Florence, Italy; 4 Schulze Center for Novel Therapeutics, Division of Oncology Research, Department of Oncology, Mayo Clinic, Rochester, MN 55905 USA

**Keywords:** Melanoma, GLI1, CX3CL1, Immune escape, Myeloid-derived suppressor cells, Dendritic cells

## Abstract

**Background:**

Melanoma progression is based on a close interaction between cancer cells and immune cells in the tumor microenvironment (TME). Thus, a better understanding of the mechanisms controlling TME dynamics and composition will help improve the management of this dismal disease. Work from our and other groups has reported the requirement of an active Hedgehog-GLI (HH-GLI) signaling for melanoma growth and stemness. However, the role of the downstream GLI1 transcription factor in melanoma TME remains largely unexplored.

**Methods:**

The immune-modulatory activity of GLI1 was evaluated in a syngeneic B16F10 melanoma mouse model assessing immune populations by flow cytometry. Murine polymorphonuclear myeloid-derived suppressor cells (PMN-MDSCs) were differentiated from bone marrow cells and their immunosuppressive ability was assessed by inhibition of T cells. Conditioned media (CM) from GLI1-overexpressing mouse melanoma cells was used to culture PMN-MDSCs, and the effects of CM were evaluated by Transwell invasion assay and T cell inhibition. Cytokine array analysis, qPCR and chromatin immunoprecipitation were performed to explore the regulation of CX3CL1 expression by GLI1. Human monocyte-derived dendritic cells (moDCs) were cultured in CM from GLI1-silenced patient-derived melanoma cells to assess their activation and recruitment. Blocking antibodies anti-CX3CL1, anti-CCL7 and anti-CXCL8 were used for in vitro functional assays.

**Results:**

Melanoma cell-intrinsic activation of GLI1 promotes changes in the infiltration of immune cells, leading to accumulation of immunosuppressive PMN-MDSCs and regulatory T cells, and to decreased infiltration of dendric cells (DCs), CD8 + and CD4 + T cells in the TME. In addition, we show that ectopic expression of GLI1 in melanoma cells enables PMN-MDSC expansion and recruitment, and increases their ability to inhibit T cells. The chemokine CX3CL1, a direct transcriptional target of GLI1, contributes to PMN-MDSC expansion and recruitment. Finally, silencing of GLI1 in patient-derived melanoma cells promotes the activation of human monocyte-derived dendritic cells (moDCs), increasing cytoskeleton remodeling and invasion ability. This phenotype is partially prevented by blocking the chemokine CCL7, but not CXCL8.

**Conclusion:**

Our findings highlight the relevance of tumor-derived GLI1 in promoting an immune-suppressive TME, which allows melanoma cells to evade the immune system, and pave the way for the design of new combination treatments targeting GLI1.

**Supplementary Information:**

The online version contains supplementary material available at 10.1186/s13046-024-03138-0.

## Introduction

Melanoma is one of the most aggressive forms of skin cancer, characterized by high metastatic ability and resistance to therapy. The melanoma tumor microenvironment (TME) is very heterogeneous, consisting of a complex network of cancer cells, immune cells, fibroblasts and endothelial cells [1]. Among immune cells, myeloid-derived suppressor cells (MDSCs) are defined by their ability to inhibit immune responses, including those mediated by T cells, and are classified into polymorphonuclear (PMN-MDSCs) and monocytic MDSCs (M-MDSCs) [2, 3]. The interplay of the cellular components of TME dictates disease progression and response to therapies [4]. Thus, a better understanding of the mechanisms controlling TME dynamics and composition will help improve the management of this dismal disease.

Work from our and other groups has reported the involvement of Hedgehog-GLI (HH-GLI) signaling in melanoma [5–12]. Canonical HH-GLI signaling is initiated by the binding of HH ligands to the receptor Patched 1 (PTCH1), which releases the repression on the G protein-coupled receptor Smoothened (SMO). Active SMO transduces the signal to activate the GLI zinc-finger transcription factors [13]. Among them, GLI1 acts as a strong transcriptional activator, whereas GLI2 and GLI3 have both activator and repressor functions [14]. Recent reports have shown that the HH-GLI signaling plays a role in modulating the immune microenvironment phenotype and function, including macrophage polarity, T cell response, and cancer-associated fibroblast (CAF) composition [15–21]. However, the consequences of downstream activation of the HH-GLI pathway on the melanoma microenvironment remain unexplored.

In this study we unveil a novel immunoregulatory role of GLI1 in the melanoma TME. We show that melanoma cell-intrinsic GLI1 alters the infiltration of immune cells by increasing PMN-MDSCs and decreasing dendritic cells (DCs) in vivo. We provide evidence that GLI1 sustains PMN-MDSC expansion and recruitment through the release of the chemokine CX3CL1. Furthermore, GLI1 silencing induces the activation of human monocyte-derived dendritic cells (moDCs), a mechanism partially inhibited by blocking CCL7. These findings provide insights into the role of GLI1 in promoting immune evasion in melanoma.

## Materials and methods

### Cell lines and reagents

Murine melanoma cell lines YUMM1.7 and YUMM5.2 were purchased from LGC Standards (www.lgcstandards.com) and cultured in Dulbecco’s modified Eagle’s medium (DMEM)-F12 (Euroclone, Milan, Italy) containing 10% fetal bovine serum (FBS) (Euroclone), 1% penicillin–streptomycin (P/S) solution (Thermo Fisher Scientific, Waltham, MA, USA) and 1% non-essential amino acids (Euroclone) at 37°C in a 5% CO_2_ incubator. B16F10 cells were kindly provided by Prof. Lido Calorini (University of Florence, Italy). HEK-293T and human melanoma cells A375 were purchased from ATCC. Patient-derived SSM2c cells were obtained from a metastatic melanoma [8].  HEK-293T, A375, SSM2c and B16F10 cells were maintained in Dulbecco’s modified Eagle’s medium (DMEM) (Euroclone) containing 10% FBS, 1% P/S and 1% glutamine (Lonza, Milan, Italy) at 37°C in a 5% CO_2_ incubator. All cells were authenticated by DNA fingerprinting analysis and regularly tested by PCR to exclude Mycoplasma contamination.

### Plasmid and virus production

Lentiviruses for gene knockdown were produced in HEK-293T cells by cotransfecting lentiviral vector, dR8.74 packaging plasmid (Addgene #22036) and pMD2.G envelope plasmid (Addgene #12259). shRNA vectors used were: pLKO.1-puro (scramble, LV-c) (Addgene #8453), pLKO.1-puro-shGLI1-1 (targeting sequence 5’-CCTGATTATCTTCCTTCAGAA-3’), pLKO.1-puro-shGLI1-2 (targeting sequence 5’-CCAAACGCTATACAGATCCTA-3’) [8], pLKO.1-puro-shGli1-1 (targeting sequence 5’-CCACATCAACAGTGAGCATAT-3’) and pLKO.1-puro-shGli1-2 (targeting sequence 5’-GCATGGGAACAGAAGGACTTT-3’). Retroviruses for gene overexpression were produced in HEK-293T cells co-transfected with pBABE-puro (pBABE) or pBABE-puro-GLI1 (pBABE-GLI1) [22] with pUMVC packaging vector and pMD2.G envelope vector.

### Generation of conditioned media

To obtain conditioned media from melanoma cells, 2 × 10^5^ B16F10 or YUMM1.7 cells were seeded into p60 cell culture dishes and transduced in the presence of polybrene (8μg/mL, Sigma-Aldrich, St. Louis, MO, USA) with pBABE or pBABE-GLI1 retroviruses, whereas 2 × 10^5^ SSM2c cells were transduced with LV-c or LV-shGLI1-1 lentiviruses. After 24 h the culture medium containing viruses was replaced with fresh complete media, and after 72 h from the transduction, puromycin was added to select transduced cells. After 10 days of selection and at least three subsequent passages, performed by double PBS wash-out and cell detachment with trypsin to eliminate the potential presence of viable retroviral particles, 5 × 10^5^ B16F10 or 1 × 10^6^ YUMM1.7 cells transduced with pBABE or pBABE-GLI1, or 5 × 10^5^ SSM2c cells transduced with LV-c or LV-shGLI1-1 were seeded in complete growth media supplemented with 10% FBS. The day after or when the cell density reached approximately 80%, media were replaced with 6 mL of fresh complete media supplemented with 2.5% FBS. After 48 h, conditioned media were harvested and filtered by 0.22μm sterile PVDF syringe filters (Millipore, Burlington, MA, USA).

### Isolation of murine PMN-MDSCs and flow cytometry analysis

Bone marrow cells were flushed from femurs and tibias of healthy C57BL/6J mice and collected in sterile conditions. After red blood cell lysis by ACK lysing buffer (Lonza), 1.5 × 10^6^ cells/well were seeded in 6-well plates with 2 mL of RPMI (Euroclone) supplemented with 10% FBS, 1% P/S (Euroclone), 1% glutamine (Euroclone), 1% Hepes (Lonza), 1% sodium pyruvate (Lonza), 1% folic acid and 1% β-mercaptoethanol (Sigma-Aldrich) and differentiated with 400 ng/μL of IL-6 and GM-CSF [23, 24] (R&D Systems, Minneapolis, MN, USA) for 5–7 days. Cells (1 × 10^5^) were then harvested, resuspended in 100μL of PBS and stained for 15 min in the dark at 4°C with Viobility Fixable dye (Miltenyi Biotec, Bergisch Gladbach, Germany) and the following anti-mouse antibodies: CD45-VioGreen (Miltenyi Biotec clone REA737), CD11b-PerCP-Vio 700 (Miltenyi Biotec clone REA592), Ly6G-APC (Miltenyi Biotec clone REA526) and Ly6C-PE (Miltenyi Biotec clone REA796) [3, 25]. Cells were washed in PBS, centrifuged for 10 min at 1200 rpm, detected and measured using CytoFLEX S (BD Beckman Coulter).

### Isolation of murine T cells and flow cytometry analysis

Spleens of healthy C57BL/6J mice were dissociated, cells were filtered through a 70 μm cell strainer (BD Biosciences, San Jose, CA, USA) and mononuclear cells were separated by density gradient centrifugation using Lymphosep (density: 1.077 g/mL; Biowest, Nuaillè, France) at 500 g for 35 min. T cells were purified using Pan T Cell Isolation Kit II mouse (Miltenyi Biotec) according to the manufacturer’s instructions. Purified T cells (1 × 10^5^) were resuspended in 100μL of PBS and stained with Viobility Fixable dye (Miltenyi Biotec) to discriminate live cells and with the following anti-mouse antibodies for 15 min at 4°C: CD45-VioGreen (Miltenyi Biotec clone REA737), CD3-PE-Vio 770 (Miltenyi Biotec clone REA641), CD4-APC-Vio 770 (Miltenyi Biotec clone REA604) and CD8-PerCP-Vio 700 (Miltenyi Biotec clone REA793). Cells were washed in PBS and detected using CytoFLEX S (BD Beckman Coulter) to measure the percentage of CD4 + and CD8 + T cells [26].

### Stimulation of purified T cells

Purified T cells (1 × 10^5^) were cultured in complete medium (RPMI, 10% FBS, 1% P/S, 1% glutamine, 1% sodium pyruvate and 1% β-mercaptoethanol) and activated in 96-well plates coated with 10 μg/mL of anti-mouse anti-CD3ε antibody (Miltenyi Biotec) and 2 μg/mL of soluble anti-mouse anti-CD28 (Miltenyi Biotec). After 48 h cells were stained with Viobility Fixable dye (Miltenyi Biotec), CD3-PE-Vio 770 (Miltenyi Biotec clone REA641), CD4-APC-Vio 770 (Miltenyi Biotec clone REA604) and CD8-PerCP-Vio 700 (Miltenyi Biotec clone REA793). Subsequently, cells were fixed, permeabilized (Inside Stain Kit, Miltenyi Biotec), stained with anti-IFNγ-PE (Miltenyi Biotec clone REA638) and detected using CytoFLEX S (BD Beckman Coulter) to evaluate intracellular IFNγ production [27].

### Inhibition of T cell function by PMN-MDSCs

For T cell suppression assay, 1 × 10^5^ activated T cells were co-cultured for 48 h with PMN-MDSC not conditioned or pre-conditioned for 48 h with CM from B16F10 pBABE or pBABE-GLI1 at 1:1, 1:3 and 1:5 ratio. Co-culture experiments were performed in T cell growth media (RPMI, 10% FBS, 1% P/S, 1% glutamine, 1% sodium pyruvate and 1% β-mercaptoethanol). The percentage of dead cells was detected by flow cytometry after staining with Viobility Fixable dye and CD3 antibody (Miltenyi Biotec) [28, 29]. Furthermore, the intracellular IFNγ production by CD4 + and CD8 + cells was evaluated as described above.

### PMN-MDSC proliferation and invasion assays

For proliferation assay, PMN-MDSCs were seeded in a 24-well plate (100.000 cells/well) and cultured with CM produced from B16F10 or YUMM1.7 melanoma cells transduced with pBABE or pBABE-GLI1 diluted 1:1 with their culturing media for 72 h. To investigate the role of CX3CL1, mouse CX3CL1 neutralizing antibody (R&D Systems) or mouse isotype control IgG (R&D Systems) were added at 1.5 μg /mL to conditioned media (CM) from murine melanoma cells. After 72 h, cells were harvested and counted.

Cell invasion assay was performed using 24-well Corning transwell membranes pre-coated with Matrigel (0.4 mg/mL; BD Biosciences, New Jersey, USA). PMN-MDSCs (50,000 cells/well) were resuspended in RPMI serum-free medium supplemented with 500 μg/mL Mitomycin C (Sigma-Aldrich) and seeded over the Matrigel coating. CM obtained from B16F10 or YUMM1.7 melanoma cells transduced with pBABE or pBABE-GLI1 was used as a chemo-attractant in the bottom chamber. To assess the role of CX3CL1 in PMN-MDSC recruitment, anti-mouse CX3CL1 neutralizing antibody (R&D Systems) or mouse isotype control IgG (R&D Systems) were added at 1.5 μg/mL to chemo-attractant conditioned media in the lower chamber. After 72 h invaded cells were fixed by PFA 4%, stained with crystal violet and counted with Image J software using the mean of 5 representative fields obtained through LEICA DFC450C microscope.

### Cytokines profiling and adenosine quantification

Cytokine and chemokine concentrations (pg/mL), released by PMN-MDSCs cultured in CM for 48 h and in refreshed media for 24 h, were quantified using Milliplex mouse cytokine/chemokine magnetic bead panel (MCYTMAG-70KPX32; Merck, Darmstadt, Germany) and by Milliplex TGFβ 1,2,3 magnetic bead kit (TGFBMAG-64 K-03; Merck), using a Bio-Plex 200 Systems (Bio-Rad, Hercules, CA, USA) following the manufacturer’s instructions. Raw data were analyzed by Bio-Plex Manager Software 6.2 (Bio-Rad). The two panels consisted of the following analytes: IL-10, IL-12p70, IL-1β, IL-6, MIP-1α (CCL3), MIP-1β (CCL4), TGFβ1, TGFβ2 and TGFβ3. Adenosine concentration was determined using a fluorometric assay (MET-5090, Cell BioLabs, San Diego, USA) following the manufacturer’s instructions and quantified on the microplate reader Victor X5 (Perkin Elmer, Waltham, MA, USA).

### Human monocyte-derived dendritic cells (moDCs)

Human monocyte-derived dendritic cells (moDCs) were generated in vitro as previously described [30] from peripheral blood mononuclear cells (PBMCs) isolated from three anonymous healthy donor (HD) buffy coats. The utilization of buffy coats, not intended for standard procedures and registered with a standard code, was authorized by the Transfusion Unit at the Careggi Hospital in Florence, Italy [31].

PBMCs were collected from buffy coats by density gradient centrifugation using Pancoll (density: 1.077 g/mL; Bioclass Srl, Pistoia, Italy) at 1500 rpm for 30 min. From isolated PBMCs, CD14 + monocytes were purified by immunomagnetic selection using the CD14 MicroBeads kit (Miltenyi Biotec) eluting the cell suspension through an LS column placed on a suitable Miltenyi MACS separator, according to the manufacturer's protocol. Purified CD14 + cells were washed once in MACS buffer (PBS 1X with 0.5% bovine serum albumin and 2 mM ethylenediaminetetraacetic acid) at 1500 rpm for 5 min, and then resuspended in complete RPMI 1640 medium (10% fetal bovine serum; 1% penicillin/streptomycin; 1% sodium pyruvate; 1% L-glutamine; 1% Hepes buffer) and counted. Monocytes were plated at a density of 2 × 10^6^/mL in complete RPMI for 7 days, in the presence of 100 ng/mL of GM-CSF and 50 ng/mL of IL-4 (R&D Systems).

### Stimulation and activation analysis of moDCs

After 7 days of differentiation, moDCs were washed once at 1300 rpm for 10 min, resuspended in complete RPMI and counted. For the first set of experiments, cells were incubated at 37°C, 5% CO_2_, at a density of 1 × 10^6^ cells/mL, for 48 h, in the following conditions: 1) moDCs in complete RPMI medium, as control; 2) in complete RPMI and LV-c CM in a volume ratio of 1:1; 3) in complete RPMI and LV-shGLI1 CM (1:1). These 3 conditions were replicated with the addition of 2 μg/mL of lipopolysaccharide (LPS; Sigma-Aldrich), as positive control. In another set of experiments, conditions 1), 2) and 3) were replicated and, in addition, moDCs were also incubated for 48 h in the presence of the anti-human CCL7 antibody (anti-hCCL7, clone 36328, R&D Systems), anti-human CXCL8 antibody (anti-hCXCL8, MAB208-100, R&D Systems) or IgG isotype, both at a final concentration of 10 μg/mL, with or without 2 μg/mL LPS. After 48 h of stimulation, moDCs were analyzed for the activation phenotype by CyFlow Space flow cytometer (Sysmex Partec, Germany) being labeled for 20 min in the dark with the following fluorescent anti-human antibodies: HLA-DR-PerCP (clone L243; eBioscence, USA); CD80-FITC (clone 2D10.4; eBioscence); CD83-PE (clone HB15e; BD Pharmingen, USA); and CD86-PE (clone IT2.2; eBioscence). Flow cytometry data were acquired following standard guidelines [32] by gating labeled cells on a forward scatter (FSC) vs a side scatter (SSC) dot plot. Data were analyzed by the Sysmex Partec FloMax software.

### Immunofluorescence

After 48 h of incubations as described above, 20.000 moDCs of each condition were used for morphological analysis. Cells were seeded on positively charged glass slides (Thermo Fisher Scientific), fixed for 15 min with 4% paraformaldehyde (PFA) (Thermo Fisher Scientific), washed with PBS, and permeabilized with PBS/FBS 5%/Triton 0.3% solution for 30 min. Cell cytoskeleton F-actin was labeled with Alexa Fluor 488 Phalloidin (Cell Signaling Technology, Danvers, MA, USA) for 15 min. Next, moDCs were washed with PBS and slides were closed with glass coverslips using mounting medium containing DAPI (ProLong Gold Antifade Mountant with DAPI, Life Technologies, USA) to label cell nuclei. Images were acquired at 40 × magnification with an Olympus BX63 fluorescence microscope coupled to the CellSens Dimension software (Olympus, Japan). Cell morphological analysis was performed by using the ImageJ software, considering the following shape descriptors: cell area (μm^2^), cell perimeter (μm) and cell circularity, equal to 4π*cell area/cell perimeter^2^ (Rasband, W.S., ImageJ, U.S. National Institutes of Health, Bethesda, Maryland, USA, https://imagej.nih.gov/ij/, 1997–2018). For each condition, a range of 15–20 random fields per slide was analyzed, where each slide field is equal to an area of approximately 0.04 mm^2^.

### Quantitative real-time PCR

Total RNA was isolated using the TriPure Isolation Reagent (Roche Diagnostics, Basel, Switzerland), and subjected to DNase I treatment (Roche Diagnostics). Reverse transcription was performed using High-Capacity cDNA Reverse Transcription Kit (Applied Biosystems, Carlsbad, CA, USA). Quantitative real-time PCR **(**qPCR) amplifications were carried out at 60°C using FastStart SYBR Green Master (Roche Diagnostics) in a Rotorgene-Q (Qiagen, Hilder, Germany). Primer sequences used for qPCR are listed in Suppl. Table 1.

### Chromatin immunoprecipitation

Chromatin Immunoprecipitation (ChIP) experiments were performed using EZ-Magna ChIP A/G Kit (Millipore cat.17–10086) according to the manufacturer’s instructions, as previously reported [33]. Chromatin fragments were then immunoprecipitated at 4°C overnight using mouse anti-GLI1 (Cell Signaling Technology cat#2643) antibody and mouse anti-RNA Pol II (Millipore). Normal mouse IgG (Millipore) was used as a negative control. qPCR was performed as described above. The primers used for ChIP-qPCR were as follows (5’ to 3’): CX3CL1promF: GCTTGGTTCCTTGGCAACAT; CX3CL1promR: TATAATGGGGTTGGCTGCCTC; PTCH1promF: CTGTCAGATGGCTTGGGTTT; PTCH1promR: GCCTACCTGGGTGGTCTCTC.

### Western blot analysis

For Western blot analysis, cells were lysed in cold RIPA buffer (1% NP-40, 150 mM NaCl, 5 mM EDTA, 0.25% NaDOC, 50 mM Tris–HCL pH 7.5, 0.1% SDS) supplemented with protease and phosphatase inhibitors and processed as previously described [34]. The primary antibodies used in this study are listed in Suppl. Table 2. ChemiDoc XRS (Bio-Rad) was used for chemiluminescence detection.

### Human and mouse cytokine arrays

Conditioned media from 48 h incubation of sub-confluent cells in 2.5% FBS-containing media was applied to the Proteome Profiler Human XL Cytokine Array (cat# ARY022B, R&D System) or Proteome Profiler Mouse XL Cytokine Array (cat# ARY028, R&D System) following manufacturer’s instructions.

### In vivo B16F10 syngeneic tumor model

B16F10 cells were transduced with pBABE or pBABE-GLI1, resuspended in a mixture of 50% Matrigel (Merck, cat#CLS356234) and 50% DMEM and injected subcutaneously into both lateral flanks of adult (7–8 weeks) female C57BL/6J mice (Charles River Laboratories, Calco, Italy) (1 × 10^5^ cells/injection). Mice were checked daily for signs of illness and distress. The subcutaneous tumor size and mouse weight were measured every day. The mice were maintained under pathogen-free conditions and with free access to standard rodent chow and water. Tumor volume was calculated with the formula V = W^2^ × L × 0.5, where W represents tumor width and L the length. Animal studies were performed according to the study protocol approved by the Institutional Animal Care and the Italian Ministry of Health (authorization n. 776/2020-PR).

### Flow cytometry analysis of melanomas

Tumors were dissected and dissociated using tumor dissociating kit mouse (Miltenyi Biotec). Cells were collected, filtered by 70μm cell strainer and centrifuged for 5 min at 1000 rpm. Cell pellets (1 × 10^7^) were resuspended in 100μL of PBS and incubated with CD45 microbeads (Miltenyi Biotec) for 15 min at 4°C. After magnetic separation the eluted cells were centrifuged for 10 min at 1000 rpm. Cell pellets (1 × 10^5^) were resuspended in 100μL of PBS and stained for 15 min with Viobility Fixable dye (Miltenyi Biotec) to discriminate live cells and incubated for 15 min in the dark at 4°C with the following anti-mouse antibodies: CD45-VioGreen (Miltenyi Biotec clone REA737), CD4-APC-Vio 770 (Miltenyi Biotec clone REA604), CD8-PerCP-Vio 700 (Miltenyi Biotec clone REA793), CD11b-PerCP-Vio 700 (Miltenyi Biotec clone REA592), Ly6G-APC (Miltenyi Biotec clone REA526), CD11c-FITC (Miltenyi Biotec clone REA754), Ly6C-PE (Miltenyi Biotec clone REA796), MHC Class II-APC-Vio 770 (Miltenyi Biotec clone REA813), F4/80-PE-Vio 770 (Miltenyi Biotec clone REA126), CD3-PE-Vio 770 (Miltenyi Biotec clone REA641) NK1.1-Vio Bright B515 (Miltenyi Biotec clone 1162), NKG2D-PE (Miltenyi Biotec clone REA1175). For regulatory T cell analysis, cells were stained with Viobility Fixable dye (Miltenyi Biotec) and with the following surface antibodies: CD45, CD4, CD8, CD3, CD25-APC (Miltenyi Biotec clone REA568). Subsequently, cells were fixed and permeabilized with FOXP3 Staining Buffer Set (Miltenyi Biotec) and stained with FOXP3-PE antibody (Miltenyi Biotec clone REA788). Cells were washed in PBS, centrifuged for 10 min at 1200 rpm, detected and measured using CytoFLEX S (BD Beckman Coulter).

### Statistical analysis

Data represent mean ± SD or mean ± SEM values calculated on at least three independent experiments. No statistical methods were used for the sample size selection. P-values were calculated using Student’s t-test (two groups) or one-way analysis of variance (ANOVA) (more than two groups). Differences were considered statistically significant at *p* < 0.05. *, *p* < 0.05; **, *p* < 0.01; ***, *p* < 0.001; ****, *p* < 0.0001.

## Results

### GLI1 overexpression in melanoma cells reprograms the immune tumor microenvironment

**Fig. 1:**
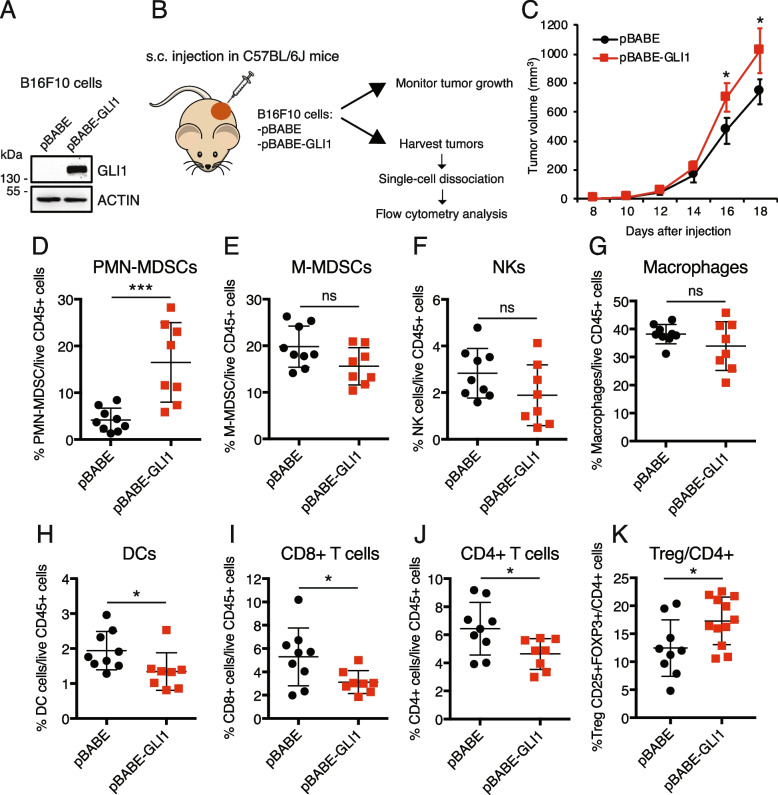
Melanoma cell-intrinsic GLI1 expression elicits profound modifications in the composition of the tumor immune infiltrate. **A** Western blot of GLI1 in B16F10 melanoma cells transduced with pBABE or pBABE-GLI1. ACTIN was used as loading control. **B** Schematic representation of the in vivo experiment. pBABE or pBABE-GLI1 B16F10 melanoma cells were subcutaneously injected into both flanks of immunocompetent C57BL/6 J mice. Following resection and single-cell dissociation of the tumor, CD45 + immune cells were characterized by flow cytometry. **C** Tumor growth of B16F10 cells transduced with pBABE or pBABE-GLI1 in C57BL/6 J mice. Data represent mean ± SEM. **D**-**K** Flow cytometry analysis of PMN-MDSCs (**D**) (*p* = 0.0008); M-MDSCs (**E**); natural killer (NK) cells (**F**); macrophages (**G**); dendritic cells (DCs) (**H**) (*p* = 0.037); CD8 + T cells (**I**) (*p* = 0.035); CD4 + T cells (**J**) (*p* = 0.030) and regulatory T cells (Treg) (**K**) (*p* = 0.027) in pBABE and pBABE-GLI1 tumors. Data represent mean ± SEM.*p < 0.05; ***p < 0.001; ns, not significant (unpaired Student *t* test)

To investigate the role of GLI1 in modulating the immune response in the melanoma TME, we used a syngeneic model of B16F10 murine melanoma cells, which express very low levels of endogenous GLI1 (Suppl. Figure 1A), in immunocompetent C57BL/6J mice. B16F10 cells were transduced with pBABE (control) or pBABE-GLI1 (GLI1-overexpressed) (Fig. 1A) and were subcutaneously injected into the flanks of mice. The degree of GLI1 overexpression using the pBABE vector is biologically feasible in melanoma cells. Indeed, it is comparable with the induction of GLI1 in cancer cells after silencing or loss of the negative regulator PTCH1 (Suppl. Figure 1B), a genetic alteration that occurs in cancer [13, 18]. After 16–18 days, tumor-infiltrating immune populations were evaluated by flow cytometry (Fig. 1B). GLI1 overexpression showed remarkable changes in the composition of the immune cell compartment, accompanied by a slight increase in tumor growth (Fig. 1C). Flow cytometry analysis revealed an increase in the percentage of PMN-MDSCs in GLI1-expressing tumors compared to controls (Fig. 1D). The frequency of infiltrating M-MDSCs, natural killer (NK) cells, and macrophages was not changed (Fig. 1E-G). Notably, the percentage of conventional dendritic cells (cDCs) was significantly decreased in GLI1-expressing tumors (Fig. 1H), as well as infiltration of CD8 + (Fig. 1I) and CD4 + T cells (Fig. 1J). In addition, the percentage of regulatory T cells (Treg) was increased in GLI1-expressing tumors (Fig. 1K). Gating strategies used for the identification of tumor infiltrating immune cells are reported in Suppl. Figure 2. These data indicate that melanoma cell-intrinsic GLI1 expression affects the composition of the primary tumor immune infiltrate, enabling an immunosuppressive TME.

### GLI1 expression in melanoma cells sustains expansion and promotes recruitment of immunosuppressive PMN-MDSCs

**Fig. 2:**
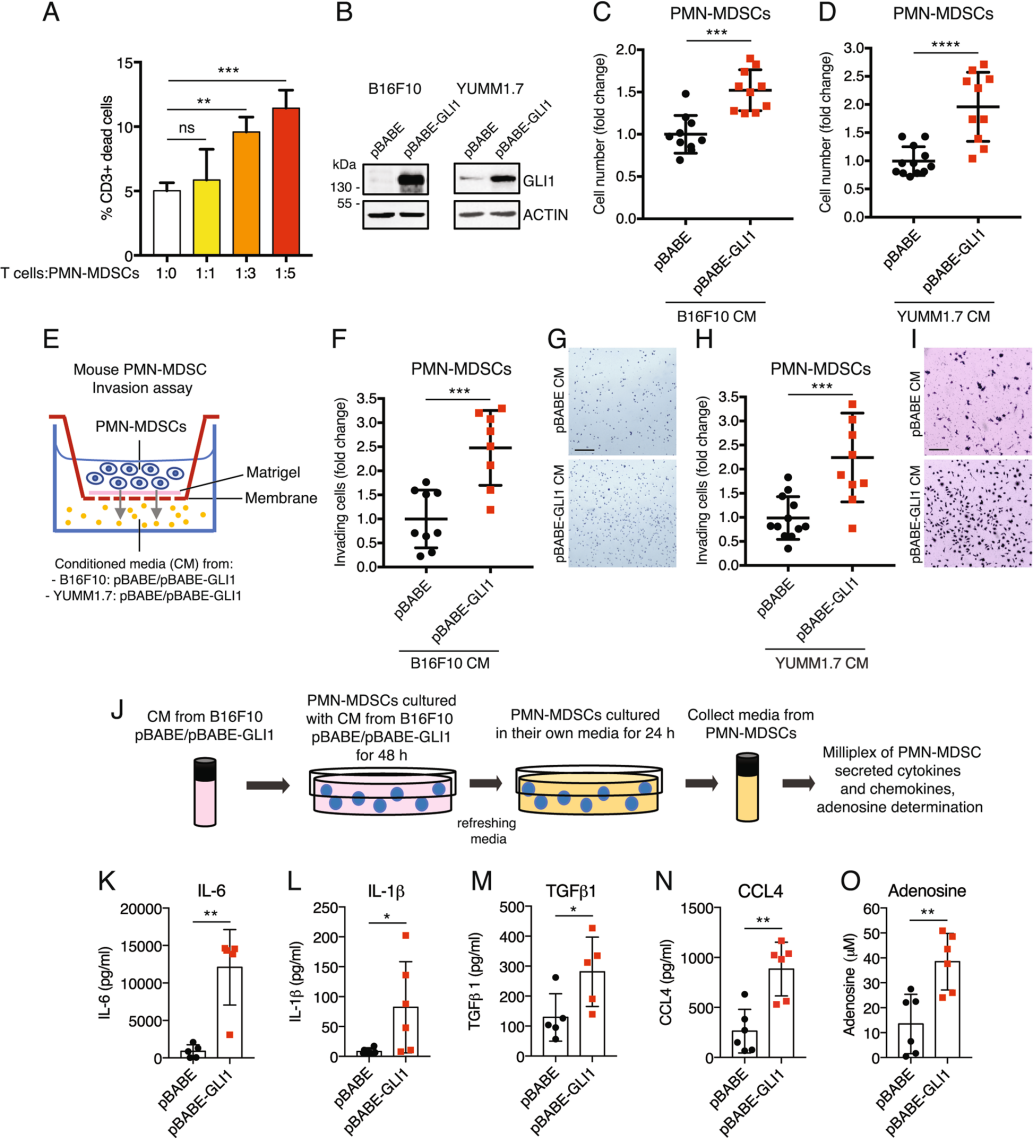
GLI1 expression in melanoma cells sustains expansion and recruitment of PMN-MDSCs in vitro. **A** Flow cytometry analysis of activated T cells and PMN-MDSCs co-cultures for 48 h at different ratios. The percentage of dead cells was determined by Viobility staining gated on CD3 + cells. Data represent mean ± SD of three independent experiments. **p < 0.01; ***p < 0.001; ns, not significant (one-way ANOVA). **B** Western blot of GLI1 in B16F10 and YUMM1.7 murine melanoma cells transduced with pBABE or pBABE-GLI1. ACTIN was used as loading control. **C, D** Proliferation assay in PMN-MDSCs cultured with CM for 72 h from B16F10 (**C**) and YUMM1.7 (**D**) cells transduced as indicated. Data represent mean ± SEM of at least three independent experiments. ***p < 0.001; **** p < 0.0001 (unpaired Student *t* test). **E** Schematic representation of PMN-MDSC invasion assay. PMN-MDSCs were seeded in the upper chamber and CM from B16F10 and YUMM1.7 cells transduced with pBABE or pBABE-GLI1 in the lower chamber. The number of invading cells was counted after 72 h. **F-I** Invasion assay in PMN-MDSCs recruited by CM from B16F10 (**F, G**) and YUMM1.7 (**H, I**) transduced as indicated. Data represent mean ± SEM of at least three independent experiments. ***p < 0.001 (unpaired Student *t* test). (**G**) and (**I**) are representative pictures of (**F**) and (**H**) respectively. **J** Schematic representation of PMN-MDSC culture conditions for cytokines and adenosine determination. PMN-MDSCs were first conditioned with CM from B16F10 cells transduced with pBABE or pBABE-GLI1 for 48 h. After refreshing, PMN-MDSCs were cultured for 24 h with their media and supernatants collected for Milliplex analysis and adenosine quantification. **K-N** Concentration (pg/mL) of cytokines IL-6 (**K**), IL-1β (**L**), TGFβ1 (**M**) and CCL4 (**N**) measured by Luminex assay in supernatants of PMN-MDSCs treated as indicated. **O** Quantification of adenosine (μM) in PMN-MDSC supernatants by fluorometric assay. Data represent mean ± SD of at least three independent experiments *p < 0.05; **p < 0.01 (unpaired Student *t* test)

As GLI1-expressing melanomas display a significant increase of PMN-MDSCs in vivo (Fig. 1D), we further investigated how GLI1 influences their behavior. To this end, bone marrow cells were isolated from C57BL/6J mice and cultured for 5–7 days in the presence of IL-6 and GM-CSF. In these conditions, we obtained a population of MDSCs with a predominance of PMN-MDSCs (CD11b + /Ly6G + /Ly6C^low^) (Suppl. Figure 3). To evaluate their immunosuppressive function, PMN-MDSCs were co-cultured with T cells isolated from spleens (Suppl. Figure 4) and activated with anti-CD3ε and anti-CD28, as demonstrated by the increase of intracellular IFNγ production (Suppl. Figure 5 and 6A-D). PMN-MDSCs co-cultured with T cells significantly increased in a dose dependent manner the percentage of dead CD3 + T cells (Fig. 2A), confirming their immunosuppressive function. PMN-MDSCs were cultured for 72 h with conditioned media (CM) derived from the two low-expressing GLI1 murine melanoma cell lines (B16F10 and YUMM1.7) (Suppl. Figure 1), transduced with pBABE or pBABE-GLI1 (Fig. 2B). CM from GLI1-expressing cells induced an expansion in the number of PMN-MDSCs compared to control CM (Fig. 2C and D). To further investigate the effect of GLI1 on PMN-MDSC recruitment, we performed an in vitro invasion assay where PMN-MDSCs were seeded over a Transwell membrane pre-coated with Matrigel (top chamber) and CM obtained from B16F10 or YUMM1.7 melanoma cells transduced with pBABE or pBABE-GLI1 were used as a chemo-attractant in the bottom chamber (Fig. 2E). The invasion of PMN-MDSCs towards GLI1-expressing CM from both cell types was significantly increased compared with the control CM after 72 h (Fig. 2F-I).

We characterized the immunosuppressive activity of PMN-MDSCs by culturing them for 48 h with CM from B16F10 cells overexpressing GLI1, refreshing the media for 24 h, and measuring the expression levels of a panel of cytokines/chemokines secreted by PMN-MDSCs (Fig. 2J). Milliplex assay showed increased levels of IL-6 and IL-1β (Fig. 2K and L), of the immunosuppressive cytokine TGFβ1 (Fig. 2M), as well as the immune cell attracting chemokine CCL4 (Fig. 2N) in GLI1-conditioned PMN-MDSCs compared to control. On the contrary, levels of CCL3 and TGFβ2 were not changed (Suppl. Figure 7D and E), whereas IL-10, IL-12p70, and TGFβ3 were below the detection limit of the assay (Suppl. Figure 7A and B). In addition, we quantified the release of adenosine by PMN-MDSCs, a ligand used by MDSCs to inhibit T cell functions in melanoma TME [35, 36]. Adenosine levels were increased in PMN-MDSCs cultured in GLI1-conditioned media compared to control (Fig. 2O; Suppl. Figure 7C), but not changed in CM of B16F10 cells transduced with pBABE or pBABE-GLI1 (Suppl. Figure 7F and G).

**Fig. 3:**
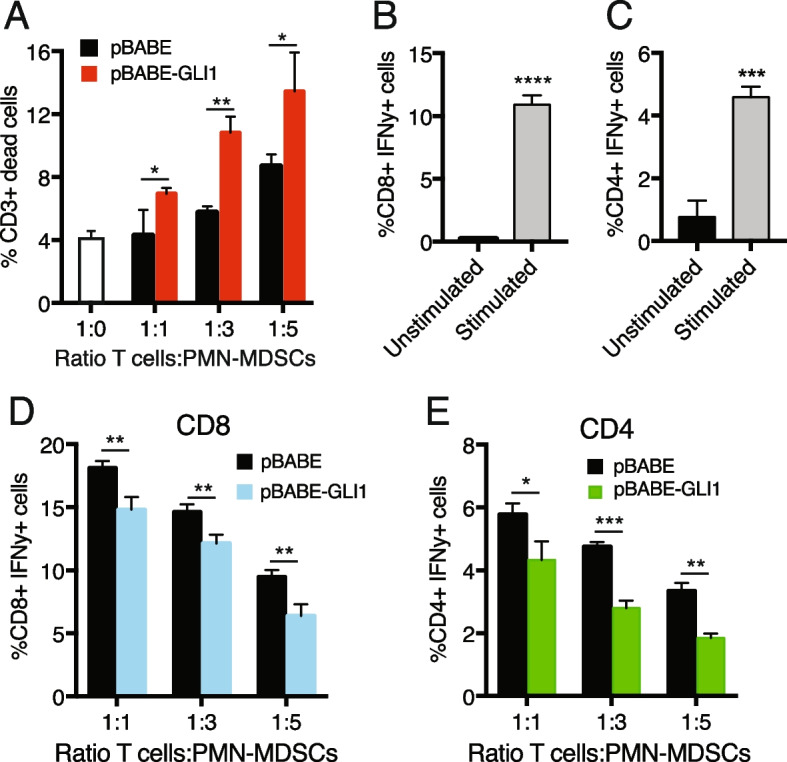
GLI1 enhances the ability of PMN-MDSCs to suppress T cells. **A** Flow cytometry analysis of activated T cells alone (white bar) or co-cultured for 48 h at different ratios with PMN-MDSCs pre-conditioned for 48 h with CM from B16F10 cells transduced with pBABE or pBABE-GLI1. The percentage of dead cells was determined by Viobility staining gated on CD3 + cells. **B**-**E** Flow cytometry analysis of CD8 + (**B**) and CD4 + (**C**) T cells activated for 48 h. The percentage of CD8 + /IFNγ + and CD4 + /IFNγ + cells was gated on CD3 + cells. **D**-**E** Flow cytometry analysis of IFNγ expression in activated CD8 + (**D**) and CD4 + (**E**) T cells co-cultured for 48 h at different ratios with PMN-MDSCs pre-conditioned for 48 h with CM from B16F10 cells transduced with pBABE or pBABE-GLI1. Data represent mean ± SD of at least three independent experiments *p < 0.05; **p < 0.01; ***p < 0.001; ****p < 0.0001 (unpaired Student *t* test)

Notably, PMN-MDSCs pre-conditioned with media from melanoma cells overexpressing GLI1 induced T cell death more efficiently than those pre-conditioned with control media (Fig. 3A). We also evaluated the capability of pBABE and pBABE-GLI1 preconditioned PMN-MDSCs to interfere with IFNγ production by CD8 + and CD4 + T cells. CD8 + and CD4 + T cells were activated with anti-CD3ε and anti-CD28, as demonstrated by the increase of intracellular IFNγ (Fig. 3B and C; Suppl. Figure 6A-D). A greater reduction of IFNγ expression was observed in CD8 + and CD4 + T cells co-cultured for 48 h with PMN-MDSCs preconditioned with pBABE-GLI1 compared to those preconditioned with pBABE CM (Fig. 3D and E; Suppl. Figure 6E).

Taken together, these results indicate that tumor-derived GLI1 plays an important role in sustaining expansion and recruitment of PMN-MDSCs, in promoting their immunosuppressive phenotype, and in suppressing CD8 + and CD4 + T cell function.

### GLI1-induced CX3CL1 contributes to PMN-MDSC expansion and recruitment

**Fig. 4:**
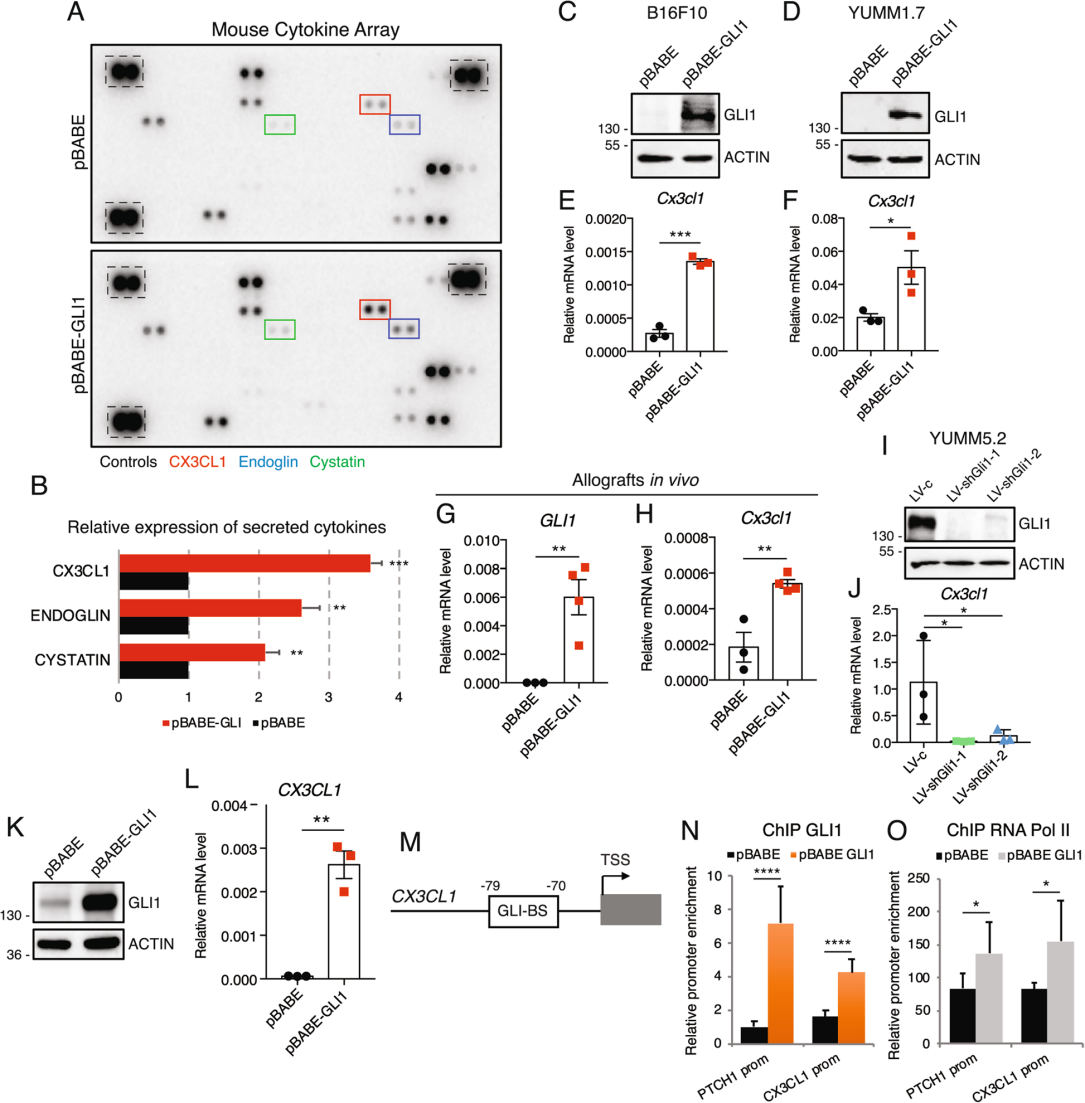
GLI1 regulates the expression of CX3CL1. **A** Representative cytokine array of the supernatants of B16F10 cells transduced with pBABE or pBABE-GLI1. **B** Expression level of cytokines that exhibited at least 1.5 fold change in pBABE-GLI1 compared to control. Protein expression was assessed by densitometric analysis using ImageJ, and each protein was normalized to array controls. The mean value of the proteins in pBABE was set to 1, and the fold change in pBABE-GLI1 was calculated for each protein. Data represent mean ± SEM. **p < 0.01; ***p < 0.001 (unpaired Student *t* test). **C, D** Western blot of GLI1 in B16F10 and YUMM1.7 cells transduced as indicated. ACTIN was used as loading control. **E, F** qPCR of *Cx3cl1* in B16F10 and YUMM1.7 cells transduced as indicated. Data represent mean ± SEM of three independent experiments. *p < 0.05; ***p < 0.001 (unpaired Student *t* test). **G, H** qPCR of *GLI1* (**G**) and *Cx3cl1* (**H**) in murine allografts injected with B16F10 cell transduced with pBABE or pBABE-GLI1. **p < 0.01 (unpaired Student *t* test). **I** Western blot of GLI1 in YUMM5.2 cells transduced as indicated. ACTIN was used as loading control. **J** qPCR of *Cx3cl1* in YUMM5.2 cells transduced as indicated. Data represent mean ± SEM of three independent experiments. *p < 0.05 (one-way ANOVA). **K** Western blot of GLI1 in human A375 melanoma cells transduced with pBABE or pBABE-GLI1. **L** qPCR analysis of *CX3CL1* in A375 cells transduced with pBABE or pBABE-GLI1. Data represent mean ± SEM of three independent experiments. **p < 0.01 (unpaired Student *t* test). **M** Schematic representation of the putative GLI consensus site in *CX3CL1* promoter. **N–O** ChIP assay showing that GLI1 and RNA Pol II bind to *CX3CL1* promoter in A375 cells. *PTCH1* promoter was used as a positive control. The y axis represents the relative promoter enrichment, normalized on the input material. Data represent mean ± SD of three independent experiments. *p < 0.05; ****p < 0.0001 (unpaired Student *t* test)

Given that chemokines and cytokines secreted in the TME play a critical role in the recruitment of immune cells [37], we analyzed the levels of 111 secreted cytokines/chemokines in the supernatant of B16F10 cells overexpressing GLI1. Their supernatants showed increased levels of CX3CL1/fractalkine, endoglin and cystatin compared to control cells (Fig. 4A). Among them, CX3CL1 showed the most significant upregulation by GLI1 (Fig. 4B). CX3CL1 is a chemokine that combines properties of chemoattractant and adhesion molecule [38, 39]. We found that GLI1 overexpression increased *Cx3cl1* mRNA levels in B16F10 and YUMM1.7 cells (Fig. 4C-F). Consistently, *Cx3cl1* mRNA levels were also increased in vivo in GLI1-expressing tumors compared to controls (Fig. 4G and H). Conversely, silencing of *Gli1* with two independent shRNAs in YUMM5.2 mouse melanoma cells, which express high levels of GLI1 (Suppl. Figure 1A), resulted in a significant reduction of *Cx3cl1* mRNA levels (Fig. 4I and J). GLI1 overexpression in human melanoma A375 cells, which express low level of GLI1 [22], increased *CX3CL1* mRNA expression (Fig. 4K and L), suggesting a direct transcriptional regulation of CX3CL1 by GLI1. Through in silico promoter analysis, we found a putative consensus GLI-binding sequence (TCCCACCCT) [40] at -79 bp to -70 bp upstream of the transcription start site of the *CX3CL1* gene (Fig. 4M). Chromatin immunoprecipitation (ChIP) assay showed an increase in GLI1 and RNA Pol II occupancy at this region in A375 (Fig. 4N and O) and HEK-293T cells (Suppl. Figure 8) overexpressing GLI1. These results confirms that GLI1 directly regulates *CX3CL1* increasing its transcription, suggesting that this cytokine may concur to the downstream effects of GLI1 in the melanoma TME.

**Fig. 5:**
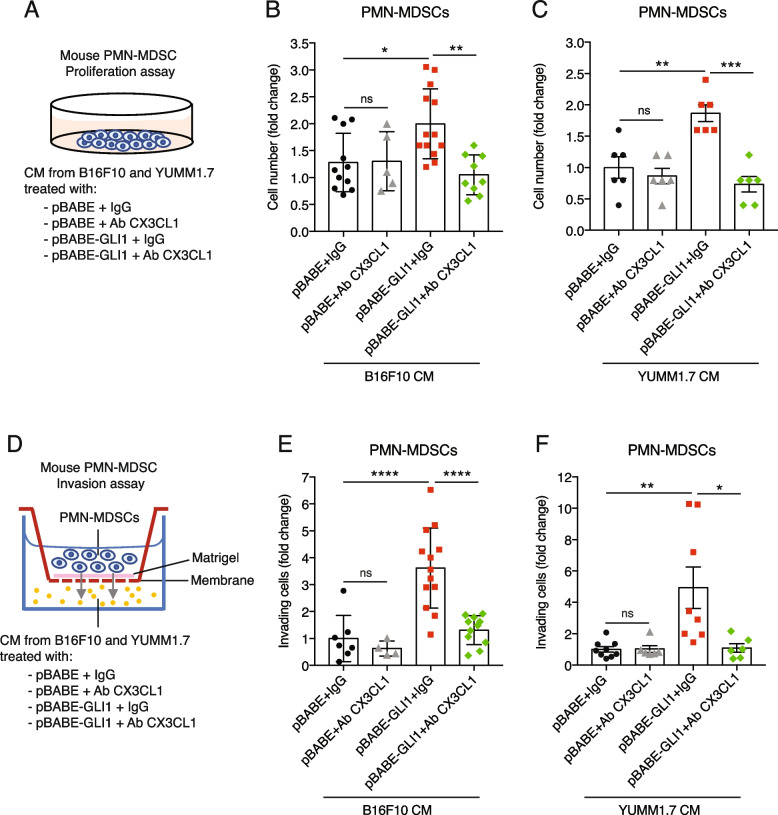
Blockage of CX3CL1 impairs GLI1-dependent expansion and invasion of PMN-MDSCs. **A** Schematic representation of PMN-MDSC culture conditioning with CM from B16F10 and YUMM1.7 cells transduced with pBABE or pBABE-GLI1 and treated with IgG or neutralizing CX3CL1 antibody (1.5 μg/mL). **B, C** Proliferation assay in PMN-MDSCs cultured 72 h with CM from B16F10 (**B**) or YUMM1.7 (**C**) cells transduced with pBABE or pBABE-GLI1 and treated with IgG or CX3CL1 antibody. **D** Schematic representation of MDSC invasion assay. PMN-MDSC were seeded in the upper chamber and CM from B16F10 and YUMM1.7 transduced with pBABE or pBABE-GLI1 and treated with IgG or CX3CL1 antibody in the lower chamber. The number of invading cells was counted after 72 h. **E, F** Invasion assay in PMN-MDSCs recruited after 72 h by CM from B16F10 (**E**) and YUMM1.7 (**F**) cells transduced with pBABE or pBABE-GLI1 and treated with IgG or CX3CL1 antibody. Data represent mean ± SD of at least three independent experiments. *p < 0.05; **p < 0.01; ***p < 0.001; ****p < 0.0001; ns, not significant (one-way ANOVA)

Notably, CX3CL1 blockade with a neutralizing antibody (Suppl. Figure 9) abrogated the increase in PMN-MDSCs proliferation (Fig. 5A-C) and invasion (Fig. 5D-F) induced by both B16F10 and YUMM1.7 CM expressing GLI1. Collectively, these results suggest that CX3CL1 mediates the expansion and recruitment of PMN-MDSCs induced by GLI1.

### GLI1 silencing in human melanoma cells promotes activation of dendritic cells

**Fig. 6:**
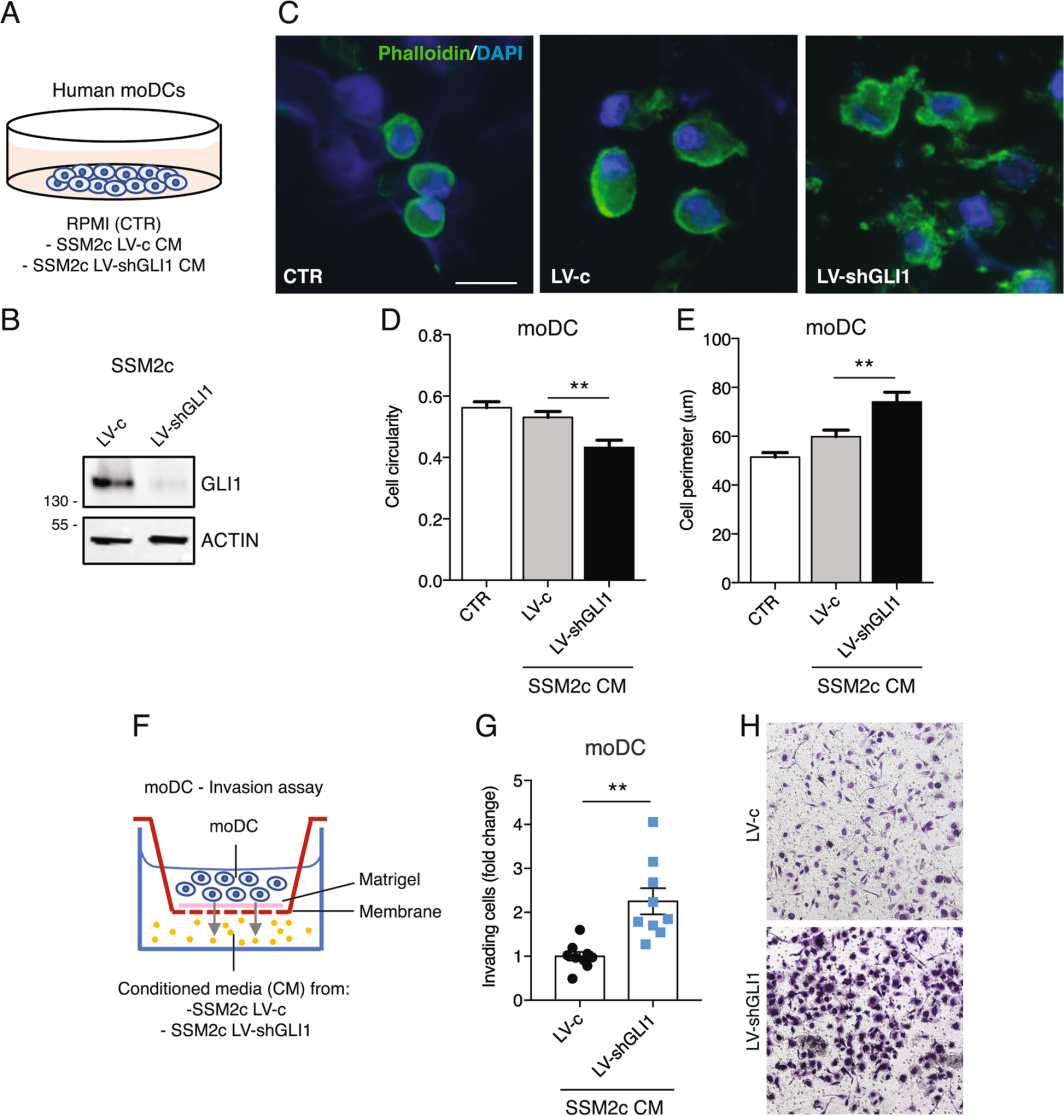
GLI1 silencing in human melanoma cells promotes the recruitment of moDCs. **A** Schematic representation of human moDCs cultured with CM from patient-derived SSM2c melanoma cells transduced with LV-c or LV-shGLI1. **B** Western blot of GLI1 in SSM2c cells transduced as indicated. ACTIN was used as loading control. **C** Representative immunofluorescence images of moDCs cultured 48 h in RPMI media (CTR), CM from SSM2c melanoma cells transduced with LV-c or LV-shGLI1. moDCs were stained with Phalloidin and counterstained with DAPI. Scale bar = 20 μm. **D, E** Cell circularity (4π*area/perimeter^2^) (**D**) and cell perimeter (μm) (**E**) analysis of moDCs treated as indicated and quantified by ImageJ. An average of 20 cells from 10–15 random glass slide areas was analyzed. Data represent mean ± SEM from three donors of three independent experiments. **F** Schematic representation of moDC invasion assay. Cells were seeded in the upper chamber and CM from SSM2c cells transduced with LV-c or LV-shGLI1 in the lower chamber. The number of invading cells was counted after 48 h. **G, H** Invasion assay in DCs recruited after 48 h by CM from SSM2c cells transduced with LV-c or LV-shGLI1. **H** Representative pictures of (**G**). Data represent mean ± SD of at least three independent experiments. **p < 0.01 (unpaired Student *t* test). DAPI = 4’, 6-diamidino-2-phenylindole

Having shown that GLI1-expressing tumors present a significant decrease in the percentage of CD11b + /CD11c + /MHCII + cDCs compared to control tumors (Fig. 1H), we sought to investigate the role of GLI1 in modulating DC activation and recruitment. To elucidate this phenotype, we turned to human monocyte-derived dendritic cells (moDCs) isolated from healthy donors (Suppl. Figure 10A), which share phenotypical and functional features with murine cDCs (CD11b + /CD11c + /MHCII +) [41] infiltrating the melanoma TME. The activation of moDCs was functionally confirmed by the increase of IL-12p40, IL-6, IL-8, IL-10 and TNFα in the supernatants of lipopolysaccharide (LPS)-activated moDCs compared to control moDCs [42] (Suppl. Figure 10B). Human moDCs were cultured in CM from patient-derived SSM2c melanoma cells transduced with LV-c or LV-shGLI1 (Fig. 6A and B). To assess moDC phenotype, we performed morphological analysis by staining their cytoskeleton F-actin with phalloidin. The analysis demonstrated that moDCs conditioned with GLI1-depleted media (LV-shGLI1) presented a significant decrease in cellular circularity and an increase in cell perimeter with the formation of protrusions, compared to moDCs conditioned with media from cells transduced with LV-c (Fig. 6C-E). The reduced cell circularity and increased cell perimeter suggest that moDCs exposed to GLI1-depleted CM are more mobile and prone to invade. Consistent with this observation, we found that LV-shGLI1 CM significantly increased the invasion of moDCs compared GLI1-expressing CM (LV-c) (Fig. 6F-H). Furthermore, we investigated whether GLI1-silenced CM affected moDC activation. moDCs from healthy donors were cultured in complete RPMI medium (CTR), or CM derived from melanoma cells SSM2c transduced with LV-c or LV-shGLI1 and after 48 h moDCs were phenotypically characterized by flow cytometry. The percentage of the activation markers HLA-DR, CD80, CD86 and CD83 was increased in moDCs upon exposure to GLI1-depleted media, suggesting that ablation of GLI1 in melanoma cells enhances moDC activation (Suppl. Figure 11). Altogether, these data suggest that the depletion of GLI1 in melanoma cells promotes moDC recruitment and activation.

**Fig. 7:**
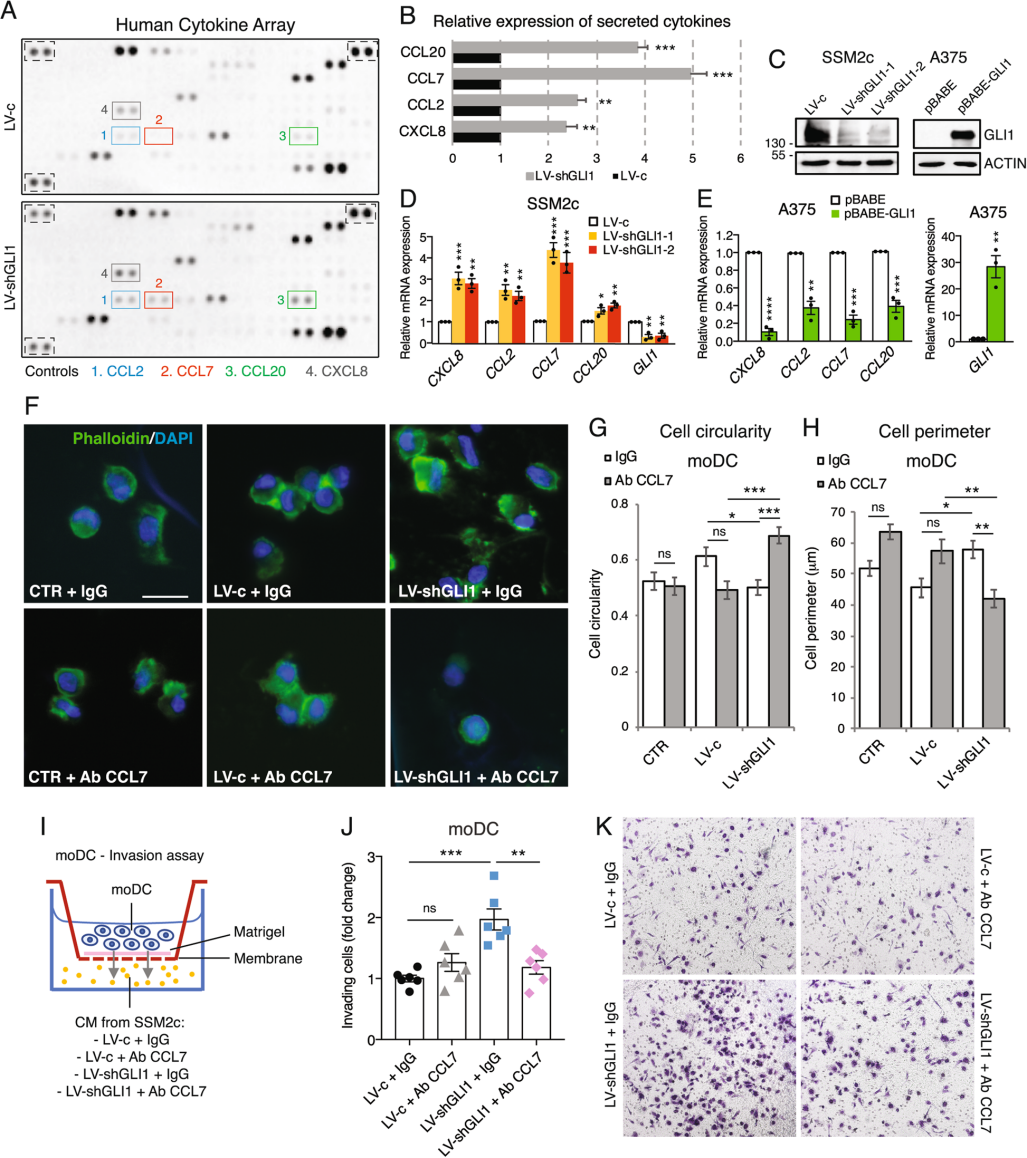
CCL7 blockade reverts the activation of moDCs promoted by GLI1 silencing in melanoma cells. **A** Representative cytokine array in supernatants of SSM2c cells transduced with LV-c or LV-shGLI1. **B** Expression level of cytokines/chemokines exhibiting at least 1.5 fold change in LV-shGLI1 compared to control (LV-c), which was equated to 1. Protein expression was assessed by densitometric analysis using ImageJ and normalized to array controls. Data represent mean ± SEM. **p < 0.01; ***p < 0.001 (unpaired Student *t* test). **C** Western blot of GLI1 in SSM2c and A375 transduced as indicated. ACTIN was used as loading control. **D-E** Validation of cytokine array with qPCR of genes shown in (**B**) in SSM2c (**C, D**) and A375 cells (**C, E**) transduced as indicated. Data are expressed as fold change relative to LV-c or pBABE, which were equated to 1. Gene expression is expressed as mean ± SEM. *p < 0.05; **p < 0.01; ***p < 0.001; ****p < 0.0001. One-way ANOVA (**D**) and unpaired Student *t* test (**E**). **F** Representative immunofluorescence images of moDCs cultured 48 h in RPMI media (CTR), CM from SSM2c cells transduced with LV-c or LV-shGLI1 and treated with neutralizing CCL7 antibody or IgG isotype matched control. moDCs were stained with Phalloidin and counterstained with DAPI. Scale bar = 20 μm. **G, H** Cell circularity (4π*area/perimeter^2^) (**G**) and cell perimeter (μm) (**H**) analysis of moDCs treated as indicated in (**F**) and quantified by ImageJ. Mean ± SEM from three donors of three independent experiments are reported. *p < 0.05; **p < 0.01; ***p < 0.001; ns, not significant (one-way ANOVA). **I** Schematic representation of moDC invasion assay. **J** Invasion assay of moDCs recruited after 48 h by CM from SSM2c cells transduced with LV-c or LV-shGLI1 and treated with blocking CCL7 antibody or IgG isotype. Data represents mean ± SEM of at least three independent experiments. **p < 0.01; ***p < 0.001; ns, not significant (one-way ANOVA). **K** Representative pictures of (**J**). DAPI = 4’, 6-diamidino-2-phenylindole

To identify secreted cytokines/chemokines modulated by GLI1 in human cells, we used SSM2c melanoma cells, which express high levels of GLI1 [22]. Cytokine array from SSM2c cells transduced with LV-c or LV-shGLI1 showed a significantly higher secretion of CCL7, CCL2, CCL20 and CXCL8 (IL-8) in CM collected from GLI1-silenced melanoma cells (Fig. 7A). The expression level of CCL7 was the most upregulated by GLI1 silencing (Fig. 7B). qPCR analysis confirmed the upregulation of *CCL7, CCL2, CCL20* and *CXCL8* mRNA level in GLI1-silenced SSM2c cells (Fig. 7C and D). To account for compensatory effects of GLI2, its expression was quantified after GLI1 silencing. No significant differences were observed in GLI2 mRNA and protein levels in SSM2c cells transduced with LV-c or LV-shGLI1 (Suppl. Figure 12), suggesting that in these cells GLI2 does not compensate for the loss of GLI1. Overexpression of GLI1 in A375 melanoma cells (Fig. 7C) drastically downregulated *CCL7, CCL2, CCL20* and *CXCL8* mRNA level (Fig. 7E). Among these cytokines, level of *CCL7* was the most affected by GLI1 modulation. The negative modulation of *Ccl7* mRNA expression by GLI1 was also confirmed in murine melanoma cells and in allografts in vivo (Suppl. Figure 13). CCL7 can exert a dual role in cancer, being able to promote or suppress tumor growth [43]. To investigate the role of CCL7 in melanoma, we tested CM from melanoma cells and found that CM recovered from GLI1-silenced cells halved the viability of SK-Mel-5, MeWo and A375 melanoma cells compared to CM from LV-c cells (Suppl. Figure 14A-C). CCL7 blocking antibody rescued melanoma cell proliferation in all three cell lines (Suppl. Figure 14A-C). In addition, a publicly available dataset from the cBioPortal for Cancer Genomics [44, 45] showed that high CCL7 expression positively correlates with overall survival of melanoma patients (*p* = 0.0029) (Suppl. Figure 14D). Altogether, these data suggest a tumor suppressive role of CCL7 in melanoma.

It was recently shown that CCL7 recruits conventional DCs to promote anti-tumor immunity in non-small cell lung cancer (NSCLC) [46]. In line with this, we found that the expression of CCL7 positively correlated with infiltration of DCs in metastatic melanoma samples (TIMER dataset *p* = 8.26e^−9^) (Suppl. Figure 14E). To assess the role of CCL7 in DC recruitment upon GLI1 silencing in melanoma cells, moDCs cultured with RPMI, or CM from SSM2c cells transduced with LV-c or LV-shGLI1 were treated with neutralizing CCL7 antibody or IgG isotype matched control. Phalloidin immunostaining showed that the CCL7 antibody abrogated the decrease of cellular circularity and the increase of cellular perimeter induced by GLI1 silencing (Fig. 7F-H). The same effect was observed in the invasion assay, where CCL7 neutralizing antibody in the lower chamber prevented DC invasion induced by GLI1-depleted CM (Fig. 7I-K). Altogether, these data highlight the role of CCL7 in GLI1-dependent DC recruitment and activation.

After activation, DCs traffic into the draining lymph node to initiate immune responses. A dramatic change in the repertoire of chemokine occurs during DC activation and maturation. Beyond CCL7, other cytokines, including CXCL8, are involved in DC migration [47]. Therefore, we tested the involvement of CXCL8, one of the cytokines whose expression was most affected by GLI1 modulation (Fig. 7D and E). Phalloidin immunostaining showed that blocking CXCL8 antibody did not rescue the decrease of cell circularity and the increase of cell perimeter induced by GLI1-depleted conditioned media (Suppl. Figure 15A-C). In addition, CXCL8 neutralizing antibody did not prevent moDC invasion induced by GLI1-depleted CM (Suppl. Figure 15D-F). Altogether, there data exclude the involvement of CXCL8 in GLI1-dependent moDC recruitment and activation.

**Fig. 8:**
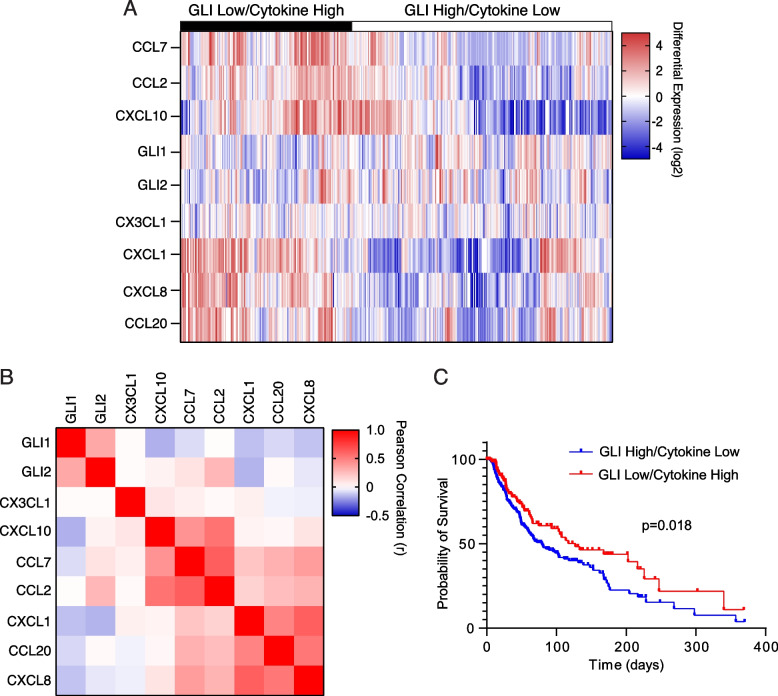
An immune signature associated with high GLI and low cytokine expression predicts poor survival in patients with cutaneous melanoma. **A** Hierarchical clustering of cutaneous melanoma patients based on GLI and cytokine expression (*n* = 443 patients, TCGA Pan-Cancer Atlas). **B** Pearson correlation of RNA expression of *GLI1, GLI2* and cytokines *CX3CL1, CXCL10, CCL7, CCL2, CXCL1, CCL20,* and *CXCL8*. **C** Kaplan–Meier survival curves for probability of survival comparing GLI High/Cytokine Low (*n* = 274) (blue line) and GLI Low/Cytokine High (*n* = 156) (red line) groups (*p* = 0.018)

Our data indicate that GLI1 negatively modulates the expression of CCL7, CCL2, CCL20 and CXCL8 (Fig. 7D and E), and of CXCL1 and CXCL10 (Suppl. Figure 16), whose expression is below the sensitivity of the cytokine array. To further support the biological relevance of these findings, we interrogated RNAseq data on the cutaneous melanoma dataset from the Pancancer Atlas [48]. Hierachical analysis showed that melanoma samples clustered in two groups; one with low GLI1 and GLI2 expression and high cytokine expression (CCL7, CCL2, CCL20, CXCL8, CXCL1 and CXCL10) (GLI Low/Cytokine High), the other group with high GLI1 and GLI2 and low cytokine expression (GLI High/Cytokine Low) (Fig. 8A and B). Of note, melanoma cases expressing GLI High/Cytokine Low presented a significantly decreased survival compared with cases with GLI Low/Cytokine High (p = 0.018) (Fig. 8C). These data suggest that high GLI levels combined to low expression of the above-mentioned cytokines identifies a subgroup of melanoma patients with decreased survival rate, supporting the immunosuppressing function of GLI1 in the TME.

## Discussion

**Fig. 9:**
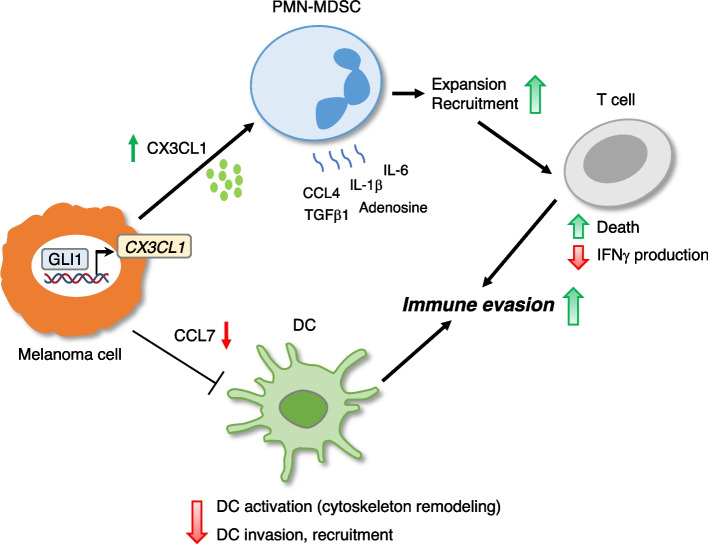
Mechanisms of immune evasion promoted by GLI1 in melanoma. GLI1 produced by melanoma cells induces transcriptional activation of the chemokine CX3CL1, which contributes to PMN-MDSC expansion and recruitment, increasing T cell death and decreasing IFNγ production. Immunosuppressive activities can be also exerted by PMN-MDSCs through increased release of IL-6, IL-1β, TGFβ1, CCL4, and adenosine. High expression of GLI1 in melanoma cells can also decrease levels of CCL7 with consequent reduced activation and recruitment of dendritic cells (DC) and enhanced immunosuppressive activity

It is well documented that the composition of the TME impacts on cancer progression and response to therapies [49, 50]. Understanding the underlying cellular and molecular mechanisms governing the interactions within the TME holds the potential to improve responses to treatment. Recent reports demonstrated that modulation of the HH-GLI pathway can alter the composition of immune cell types within the TME in a context-dependent manner [15–21, 51]. For instance, Hedgehog signaling has been shown to promote tumor-associated macrophage polarization and metabolism [17, 19]. Conversely, inhibition of the Hedgehog pathway in pancreatic cancer affects CAF composition, which leads to a decrease in cytotoxic T cells and expansion in regulatory T cells, consistent with increased immunosuppression [20]. However, the impact of the transcription factor GLI1 on tumor-associated immune cells is still unexplored in melanoma. Our investigations reveal that GLI1 elicits profound effects in the composition of tumor-infiltrating immune cells, mainly characterized by the enrichment of immunosuppressive PMN-MDSCs and Treg, and reduction of immune-stimulant DCs and CD8 + and CD4 + T cells, providing insights into a novel role for GLI1 in shaping melanoma immune escape (Fig. 9). In addition, our study allows the identification of an immunosuppressive signature characterized by high GLI and low cytokine/chemokine expression (CCL7, CCL2, CCL20, CXCL8, CXCL1 and CXCL10), which could be used to stratify melanoma patients with poor survival.

Recent reports have highlighted the significance of MDSCs in melanoma progression, given their ability to foster an immunosuppressive TME and promote resistance to immunotherapy [52]. Our study identifies GLI1 as a potential player in PMN-MDSC recruitment in the TME. Tumor-derived GLI1 sustains expansion and invasion of PMN-MDSCs in vitro, and promotes their immunosuppressive activity on T cells, inhibiting their effector capability, as demonstrated by the reduced production of IFNγ by CD4 + and CD8 + T cells. Moreover, GLI1 induces an increased release of immunosuppressive cytokines (IL-6, IL-1β, TGFβ1, and CCL4) [53–56] and of adenosine from PMN-MDSCs. Adenosine is a nucleotide generated from hydrolysis of AMP, which inhibits the activation and effector functions of T cells [37, 38]. Furthermore, CCL4 could be involved in the recruitment of Treg observed in vivo [57]. Altogether these factors reinforce the immunosuppressive behavior of GLI1 in melanoma TME.

The investigation of the molecular mechanism underlying GLI1-mediated PMN-MDSC expansion and invasion uncovered the role of CX3CL1. Our data demonstrate the direct transcriptional regulation of CX3CL1 by GLI1 in melanoma cells, consistent with a recent study [58]. The finding that the blockade of CX3CL1 reverts the increase in PMN-MDSC proliferation and invasion induced by GLI1, suggests that this phenotype could be mediated by increased expression and secretion of CX3CL1 by melanoma cells.

Another finding of this study is the decreased infiltration of cDCs in GLI1-expressing tumors. This result is consistent with a previous work, in which pharmacological inhibition of SMO with Vismodegib led to increased infiltration of dendritic cells within the TME in an immunocompetent mammary cancer model [16]. Murine cDCs can be classified into two main subsets; conventional type 1 dendritic cells (cDC1), which express CD11c and MHC-I, and conventional type 2 dendritic cells (cDC2) characterized by the expression of the surface markers CD11b, CD11c, and MHC-II [59]. Notably, the cDC population identified in our melanoma model resembles the cDC2 subset, which is specialized for priming of anti-tumor CD4 + T cell immunity [60]. There is also evidence that cDC2 and moDCs share phenotypic and functional features, including cross-present antigen [59]. Therefore moDCs were used for further functional in vitro assays.

Our investigations show that moDCs cultured in GLI1-depleted CM undergo a reorganization of the actin cytoskeleton, with a significant decrease in cell circularity and increase in cell perimeter with the formation of dendritic protrusions. Cytoskeleton exerts a role in several moDC functions, including the interaction between DCs and T cells. A correct moDC cytoskeleton organization determines stability and duration of cell contacts that are crucial in the formation of an efficient moDCs/T immune synapse [61]. We may hypothesize that the altered cytoskeletal organization that we observed disturbs the efficient formation of the immune synapse, affecting the following T cell activation. Furthermore, this phenotype endows moDCs with enhanced invasion capability, suggesting that lack of GLI1 in CM induces chemokines or soluble factors that promote activation of moDCs. In the attempt to elucidate the factors involved in this process, we identify CCL7 as one of the chemokines secreted by GLI1-silenced melanoma cells. CCL7 binds to CCR1, CCR2, and CCR3 receptors, which are mainly expressed on the surface of antigen-presenting cells, including DCs [62]. Our data show that the blockade of CCL7 partially reverts the decrease in cell circularity, the increase in cell perimeter and the increased invasion of moDCs induced by GLI1 silencing in CM, suggesting that GLI1 inhibits CCL7 to reduce DC activation and recruitment (Fig. 9). At the moment it is unclear whether CCL7 is directly modulated by GLI1. Nevertheless, bioinformatic analysis did not identify any canonical GLI consensus sequence in the promoter of CCL7, suggesting an indirect regulation. Besides CCL7, other chemokines are involved in the trafficking of DCs from the periphery toward lymph nodes. Among them, CXCL8, which is one of the most affected by GLI1 modulation, plays a role in the recruitment of DCs [47]. However, our in vitro data suggest that CXCL8 is not involved in GLI1-dependent moDC recruitment and activation.

Overall, this study demonstrates the role of GLI1 in promoting an immunosuppressive TME, which allows melanoma cells to evade the immune system. Our findings show that expression of GLI1 in melanoma cells not only sustains the expansion and recruitment of pro-tumorigenic PMN-MDSCs and skews them towards an immunosuppressive phenotype, but also diminishes anti-tumorigenic cDC populations. The reduced recruitment of cDCs and increased infiltration of PMN-MDSCs in the TME could be responsible for the decreased infiltration of CD8 + and CD4 + T cells observed in vivo. The GLI1-driven mechanisms of immunosuppression identified in this study could provide insights into the design of new treatments targeting GLI1 in combination with immunotherapy.

### Supplementary Information

Supplementary Material 1.

## Data Availability

The data generated in this study are available within the article and its supplemental data files. Raw data and datasets used and/or analyzed during the current study are available from the corresponding author upon reasonable request.

## Abbreviations

CAF: Cancer-associated fibroblast
ChIP: Chromatin Immunoprecipitation
CM: Conditioned media
DCs: Dendritic cells
HH: Hedgehog
MDSCs: Myeloid-derived suppressor cells
moDCs: Monocyte-derived dendritic cells
PBMCs: Peripheral blood mononuclear cells
PMN-MDSCs: Polymorphonuclear myeloid-derived suppressor cells
PTCH1: Patched 1
SMO: Smoothened
TME: Tumor microenvironment
