# Supplementary material for: Tumor-derived GLI1 promotes remodeling of the immune tumor microenvironment in melanoma
Source: J Exp Clin Cancer Res. 2024 Aug 2;43:214. doi: 10.1186/s13046-024-03138-0 (PMC11295348; doi:10.1186/s13046-024-03138-0)
Supplement: Supplementary file 1 — Supplementary Material 1. [file 13046_2024_3138_MOESM1_ESM.docx]

**Supplemental Information**

**Tumor-derived GLI1 promotes remodeling of the immune tumor microenvironment in melanoma**

Alessandro Giammona^1^^, Chiara De Vellis^1^^, Enrica Crivaro^1,2^^, Luisa Maresca^1,3^, Roberta Amoriello^3^, Federica Ricci^1^, Giulia Anichini^1^, Silvia Pietrobono^1^, David R. Pease^4^,

Martin E. Fernandez-Zapico^4^, Clara Ballerini^3^, Barbara Stecca^1*^

**SUPPLEMENTAL MATERIALS AND METHODS**

**Human melanoma cell lines**

A375, MeWo and SK-Mel-5 human melanoma cells were purchased from ATCC. Cells were maintained in Dulbecco’s modified Eagle’s medium (DMEM) (Euroclone, Milan, Italy) containing 10% FBS, 1% P/S and 1% glutamine (Lonza, Milan, Italy) at 37°C in a 5% CO_2_ incubator. All cells were authenticated by DNA fingerprinting analysis and regularly tested by PCR to exclude Mycoplasma contamination.

**Analysis of cytokines and chemokines concentration in moDCs supernatants**

A panel of cytokines and chemokines was analyzed in cell supernatants of unstimulated moDCs (control, CTR) or activated for 24 h with 1 mg/mL of lipopolysaccharide (LPS). moDCs were cultured at a density of 1x10^6^ cells/mL in complete RPMI 1640 medium (10% fetal bovine serum; 1% penicillin/streptomycin; 1% sodium pyruvate; 1% L-glutamine; 1% Hepes buffer) and incubated for 72 h at 37°C, 5% CO_2_. Cytokines and chemokines measurement was performed by Luminex assay (#HCYTA-60K kit, Millipore, Darmstadt, Germany), following the manufacturer’s protocol, and a Bio-Plex device (Bio-Rad, Hercules, CA, USA). The sensitivity detection limit of the kit was 1.0 pg/mL.

**Plasmids and virus production**

Lentiviruses for PTCH1 knockdown were produced in HEK-293T cells by cotransfecting lentiviral vector, dR8.74 packaging plasmid (Addgene #22036) and pMD2.G envelope plasmid (Addgene #12259). Lentiviral vectors were pLV-CTH (LV-c) and pLV-CTH-shPTCH1 (LV-shPTCH1) with targeting sequence 5’-GCACTATGCTCCTTTCCTC-3’.

**SUPPLEMENTAL FIGURES**

**
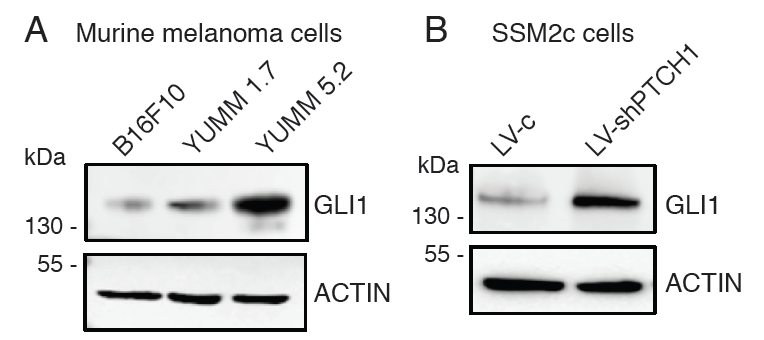
**

**Suppl. Figure 1. GLI1 expression in murine melanoma cell lines. A,** Western blot of endogenous GLI1 in B16F16, YUMM1.7 and YUMM5.2 murine melanoma cells. **B,** Western blot shows the degree of induction of endogenous GLI1 protein expression upon silencing of PTCH1 in SSM2c human melanoma cells. ACTIN was used as loading control.

**
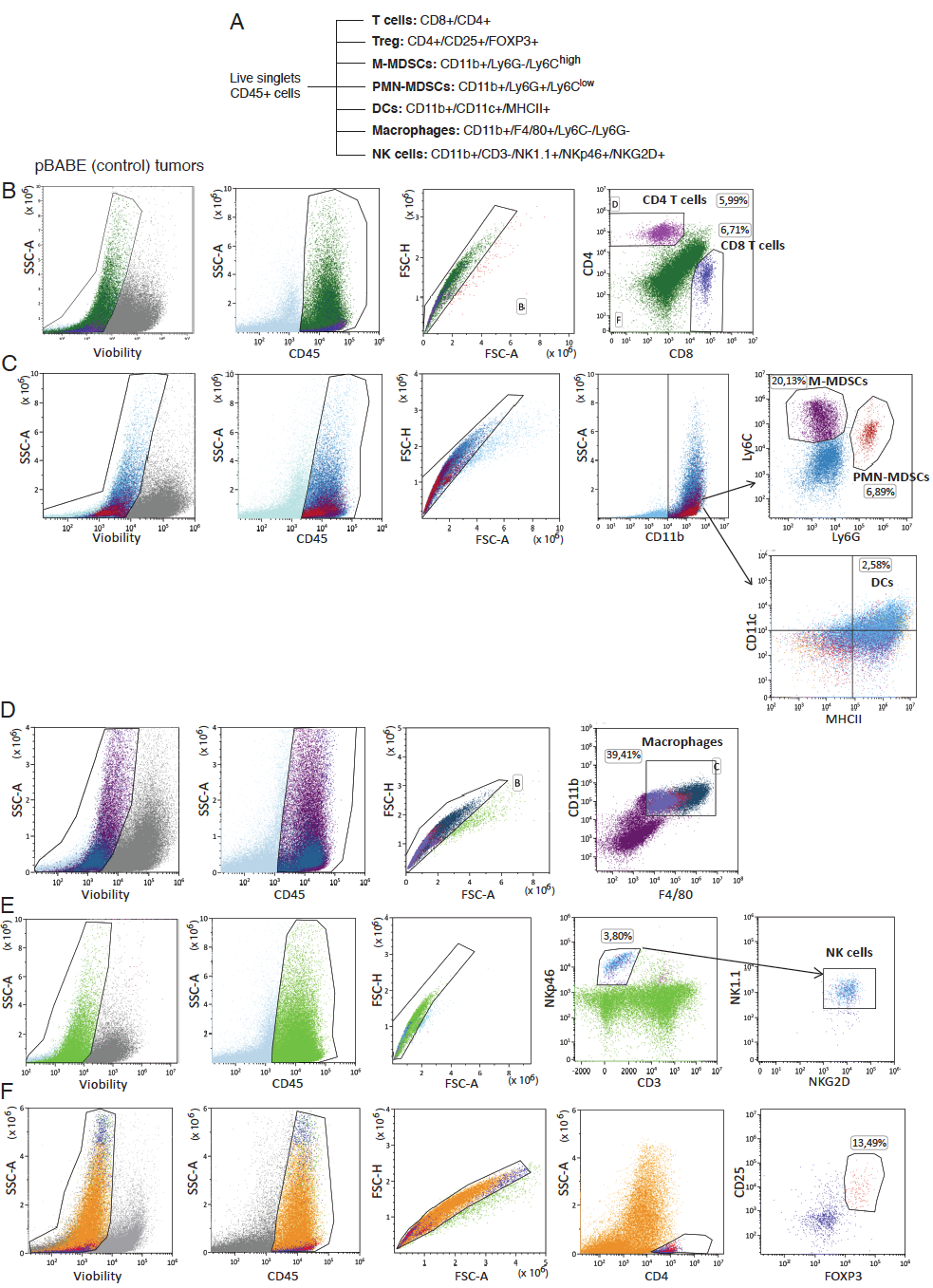
**

**
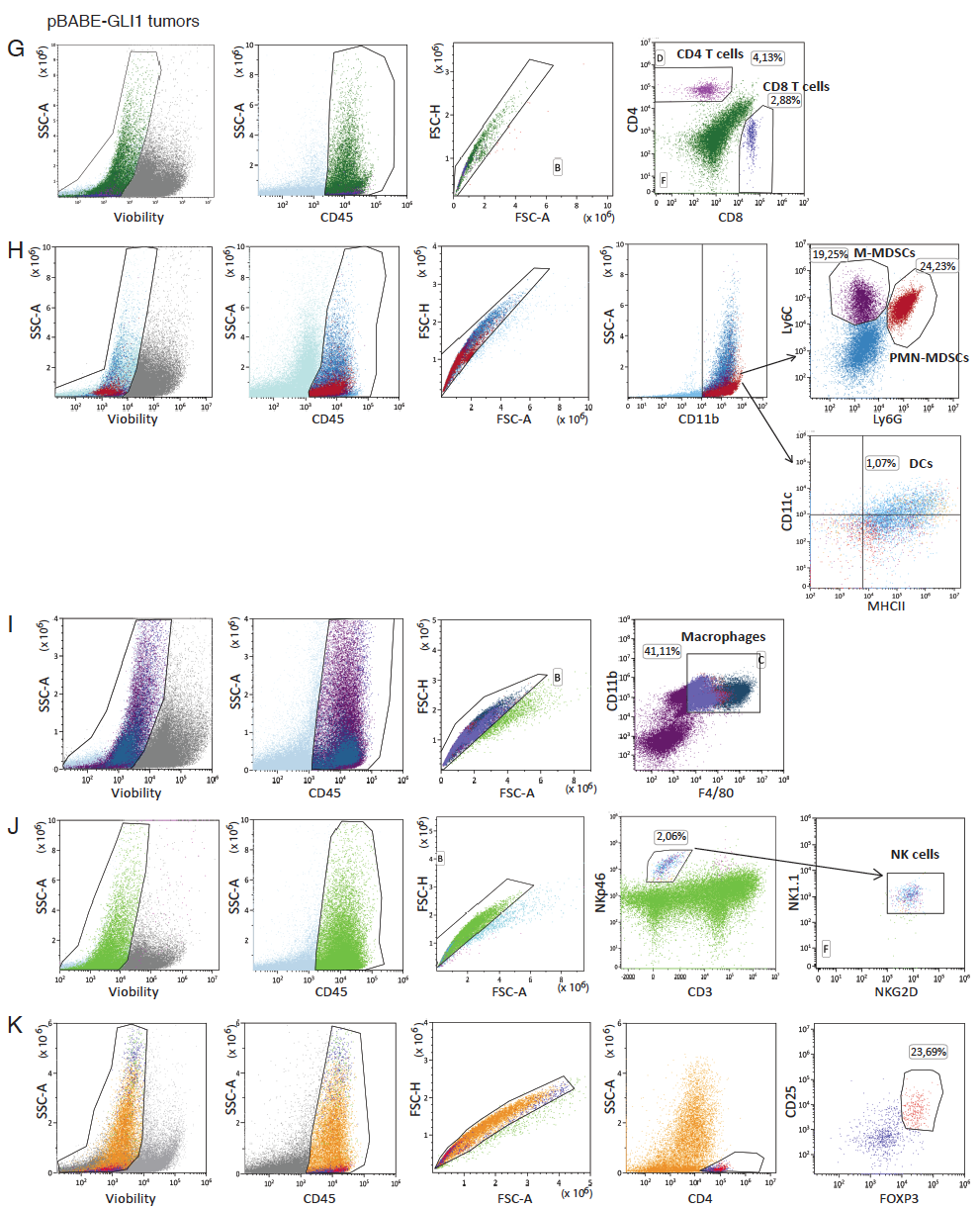
**

**Suppl. Figure 2. Flow cytometry analysis and gating strategies used for the identification of tumor infiltrating immune cells. A,** Schematic representation of markers used to identify tumor infiltrating immune populations. Following resection and single-cell dissociation of the tumor, immune cell populations were gated on CD45+ cells versus viable cells and doublets were excluded comparing FSC-H versus FSC-A. CD45+ immune cell infiltrates were assayed based on the indicated markers. **B-K,** Flow cytometry gating strategy used to determine the percentage of CD4+ and CD8+ T cells (**B,G**); polymorphonuclear myeloid-derived suppressor cells (PMN-MDSCs), monocytic myeloid-derived suppressor cells (M-MDSCs) and dendritic cells (DCs) (**C,H**); macrophages (**D,I**); natural killer (NK) cells (**E,J**) and Treg (**F,K**) from control (pBABE) (**B-F**) and GLI1-overexpressing (pBABE-GLI1) tumors (**G-K**). A representative tumor for each group (pBABE and pBABE-GLI1) is shown.

**
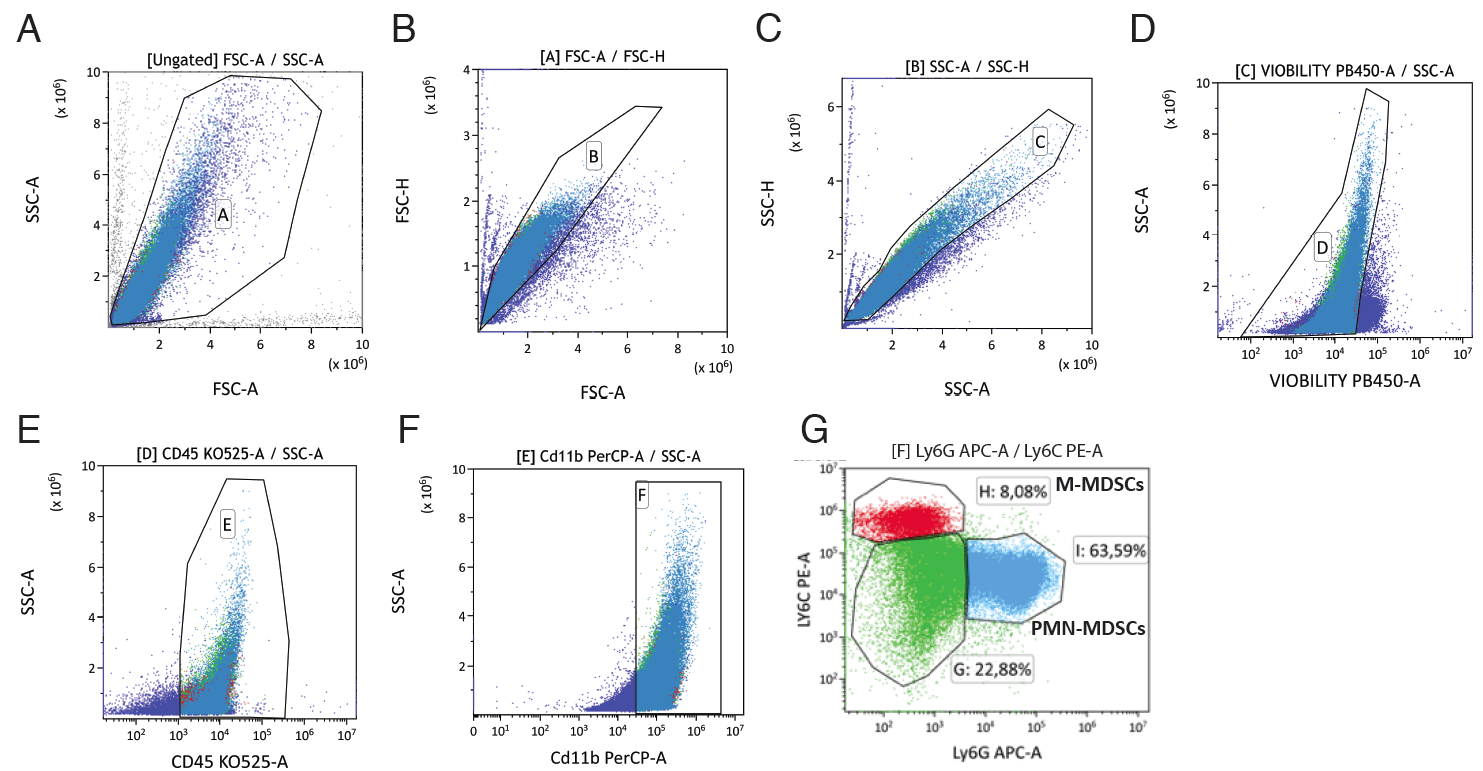
**

**Suppl. Figure 3. Flow cytometry gating strategy used to evaluate differentiation of PMN-MDSCs**. **A,** Cells were included in Gate A on the total of captured events in SSC-A (side scatter) versus FSC-A (forward scatter) plot. **B-C,** Doublets were excluded as shown in gates B and C. **D,** Live cells (gate D) were discriminated from death cells using Viobility Fixable dye. **E-F,** CD11b+ cells (**F**) were gated on CD45+ cells (**E**). **G,** Phenotype of CD45+/CD11b+ was determined by the expression of Ly6C and Ly6G markers (PMN-MDSCs: CD45+/CD11b+/Ly6G+/Ly6C^low^; M-MDSCs: CD45+/CD11b+/Ly6C^high^/Ly6G-).

**
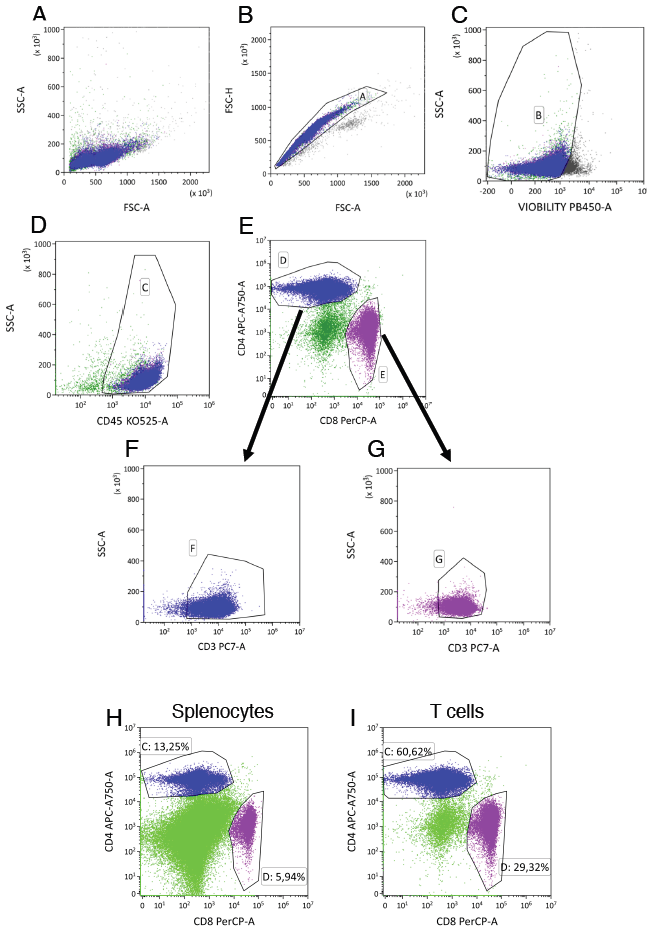
**

**Suppl. Figure 4. Lymphocyte isolation from spleens.** **A-G**, Flow cytometry gating strategy used to determine the enrichment of CD4+ and CD8+ T cells isolated from spleens. Doublets and dead cells were excluded as shown in **B** and **C**. CD4+ and CD8+ T cells (**E**) were gated on CD45+ cells (**D**). CD3 expression of CD4+ and CD8+ T cells was evaluated in **F** and **G**. **H-I**, Flow cytometry analysis of CD4+ and CD8+ T cell percentages in splenocytes (**H**) and CD4+ and CD8+ T cells purified using mouse Pan T Cell Isolation Kit II (**I**).

**
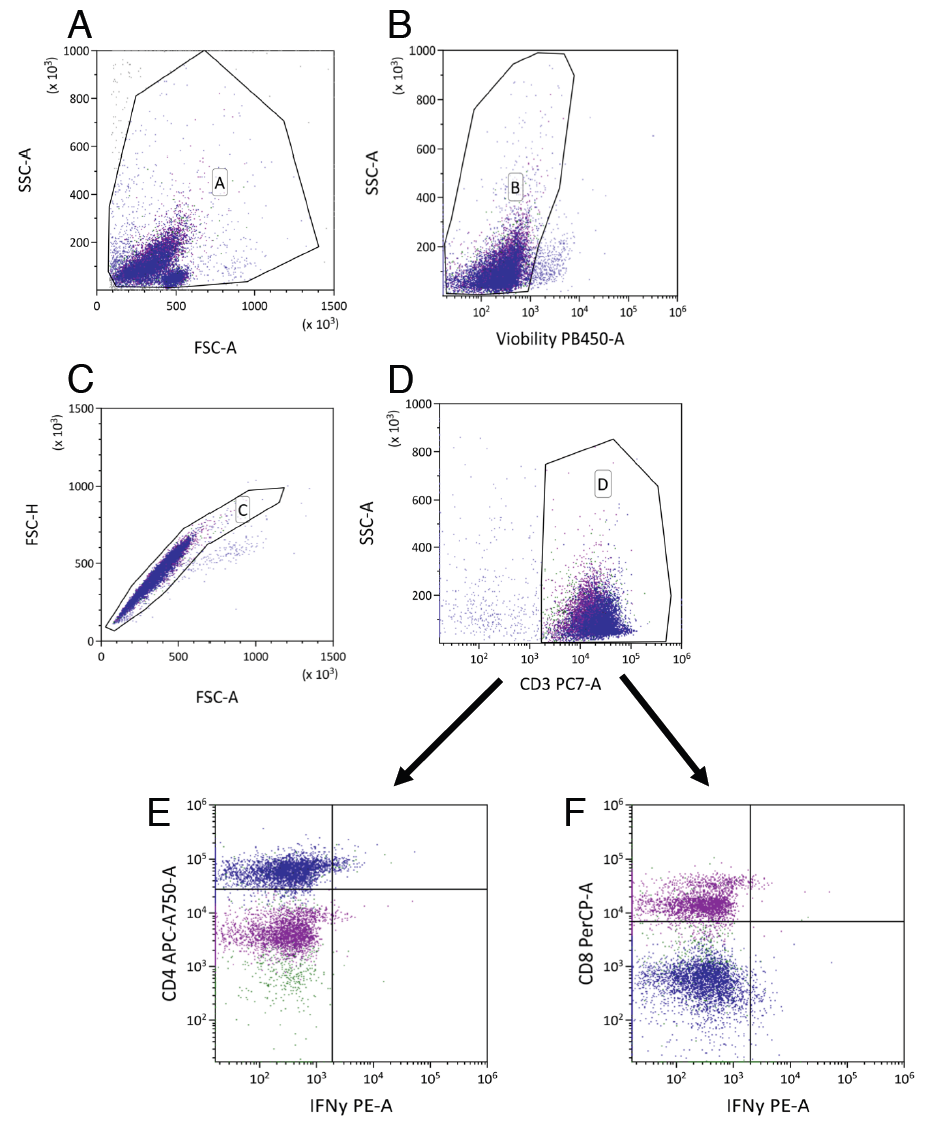
**

**Suppl. Figure 5. Flow cytometry gating strategy of intracellular IFN ** Cells were included in Gate A on the total of captured events in SSC-A (side scatter) versus FSC-A (forward scatter) plot (**A**). Dead cells and doublets were excluded as shown in **B** and **C**. The intracellular production of IFNγ in CD4+ (**E**) and CD8+ (**F**) T cells was evaluated in CD3+ cells (**D**).

**
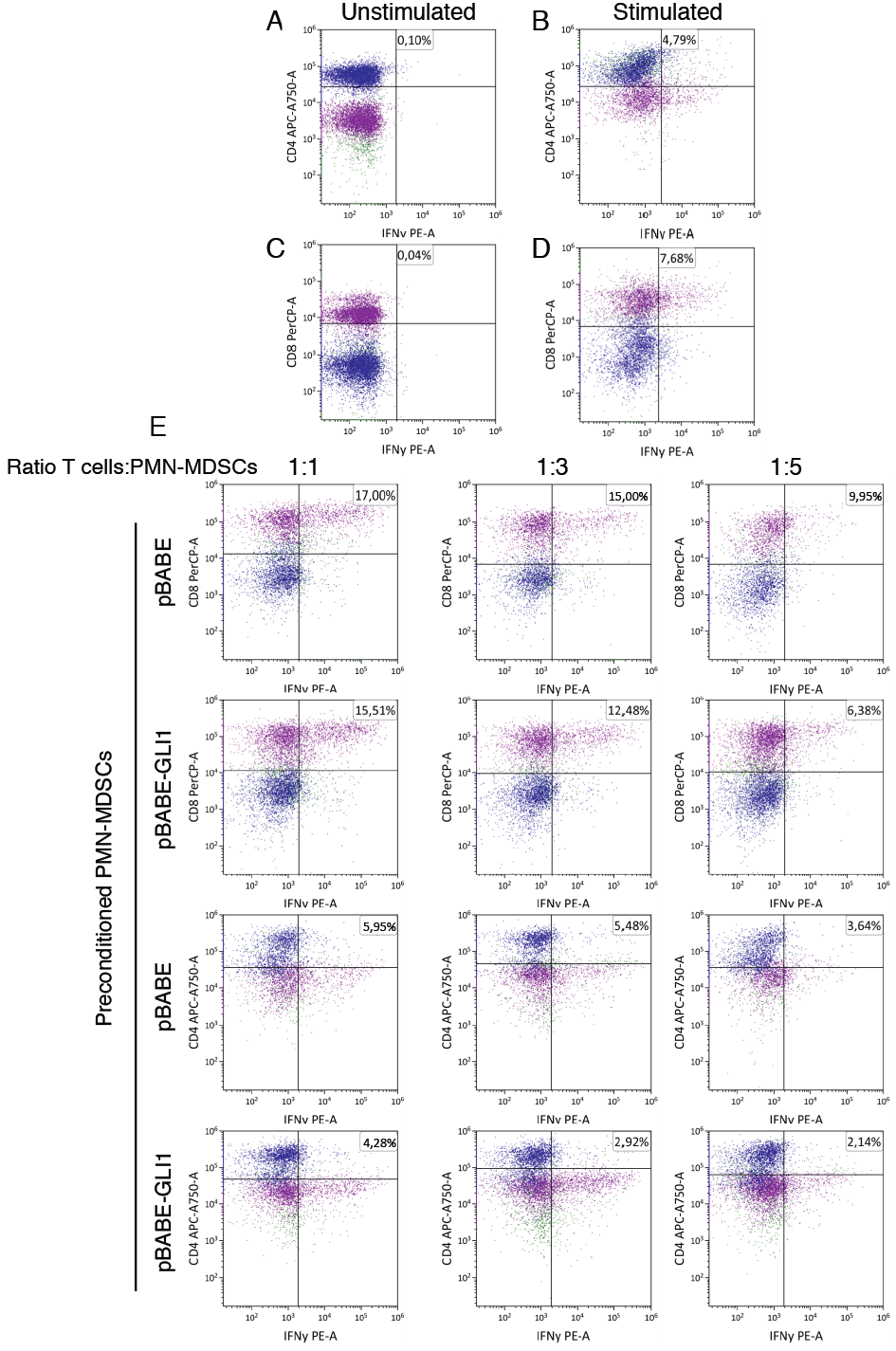
**

**Suppl. Figure 6. Flow cytometry analysis of intracellular IFN A-D,** Flow cytometry analysis of intracellular IFNγ in purified CD4+ and CD8+ T cells before (**A,C**) and after activation (**B,D**) with anti-CD3ε and anti-CD28. **E**, Flow cytometry analysis of intracellular IFNγ in purified CD4+ and CD8+ T cells co-cultured for 48 h at different ratios with PMN-MDSCs pre-conditioned for 48 h with CM from B16F10 cells transduced with pBABE or pBABE-GLI1.

**
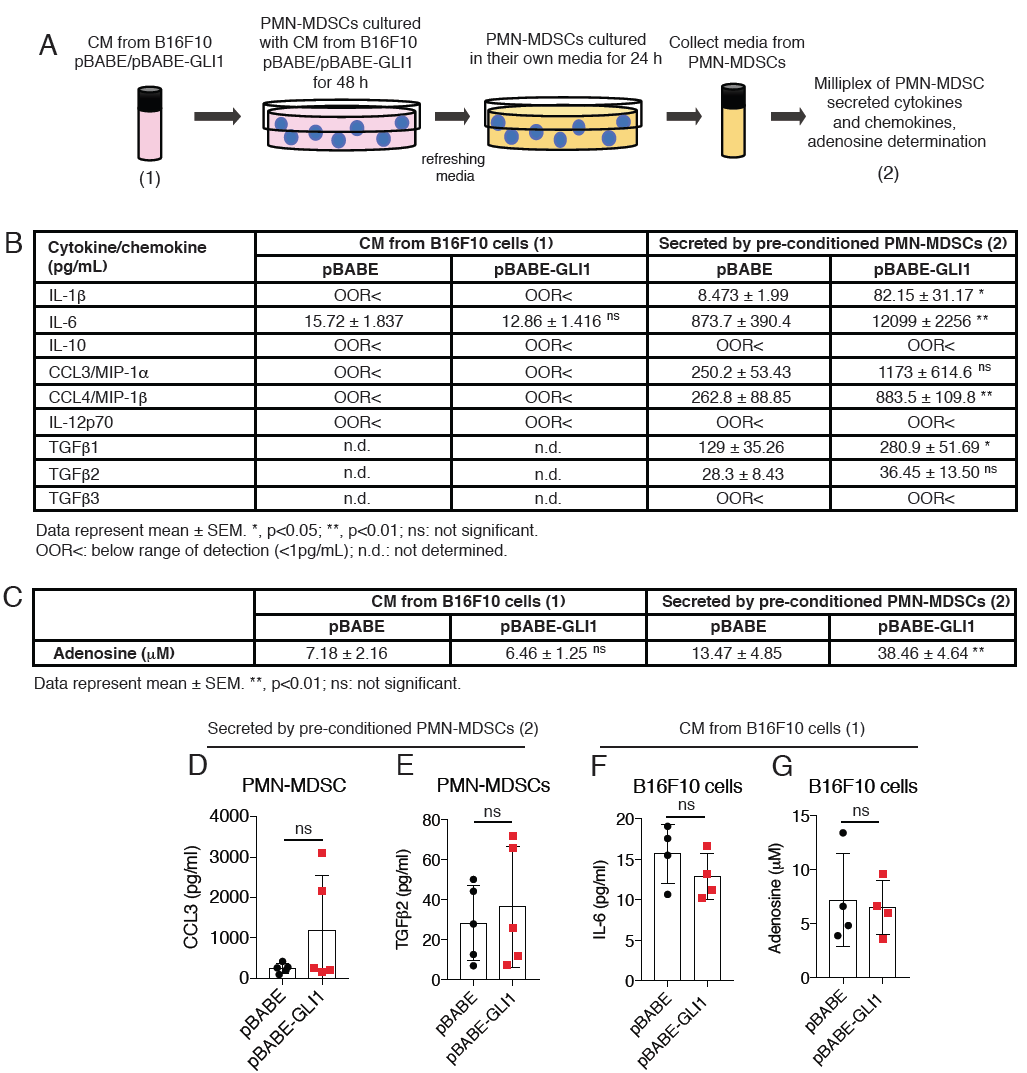
**

**Suppl. Figure 7. Quantification of cytokines/chemokines and adenosine released by B16F10 cells and pre-conditioned PMN-MDSCs. A,** Schematic representation of PMN-MDSC culture conditions for cytokines and adenosine determination. PMN-MDSCs were first conditioned with CM from B16F10 cells transduced with pBABE or pBABE-GLI1 for 48 h. After refreshing, PMN-MDSCs were cultured for 24 h with their media and supernatants collected for Milliplex analysis and adenosine quantification. **B-C**, Summary of concentration of cytokines/chemokines (pg/mL) (**B**) and adenosine (μM) (**C**) in CM from B16F10 melanoma cells transduced with pBABE or pBABE-GLI1, and secreted by pre-conditioned PMN-MDSCs (as shown in Fig. 2K-O). **D-E**, Concentration (pg/mL) of CCL3 (**D**) and TGFβ2 (**E**) secreted by pre-conditioned PMN-MDSCs. **F-G,** Concentration (pg/mL) of IL-6 (**F**) measured by Luminex assay and fluorometric quantification (μM) of adenosine (**G**) in CM from B16F10 melanoma cells transduced with pBABE or pBABE-GLI1. Data represent mean ± SEM (**B,C**) or mean ± SD (**D-G**) of at least three independent experiments. **p* < 0.05; ***p* < 0.01; ns, not significant (unpaired Student *t* test).


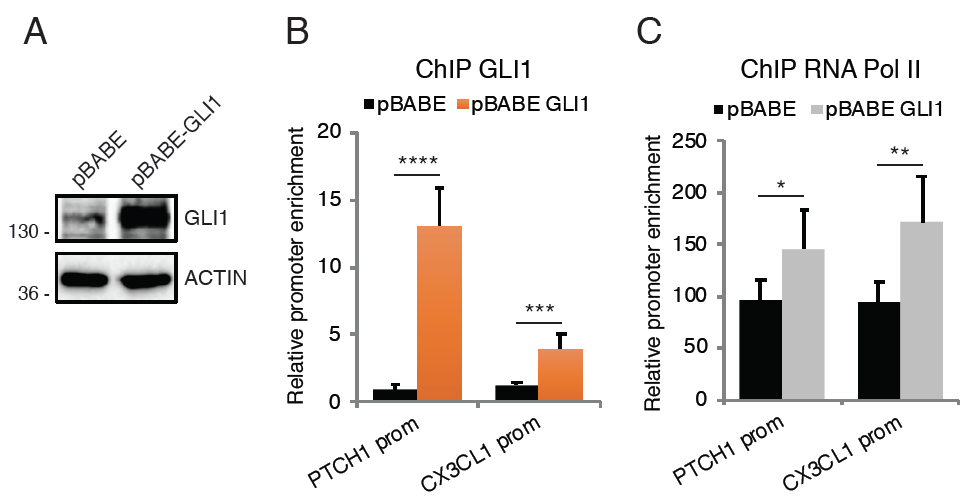


**Suppl. Figure 8. CX3CL1 is a direct target of GLI1 in HEK-293T cells.** **A,** Western blot of GLI1 in HEK-293T cells transduced with pBABE or pBABE-GLI1. ACTIN was used as loading control. **B-C,** ChIP assay showing that GLI1 and RNA Pol II bind to *CX3CL1* promoter in HEK-293T cells. *PTCH1* promoter was used as a positive control. The y axis represents the relative promoter enrichment, normalized on the input material. Data represent mean ± SD of three independent experiments. **p* < 0.05; ***p* < 0.01; ****p* < 0.001; *****p* < 0.0001 (unpaired Student *t* test).


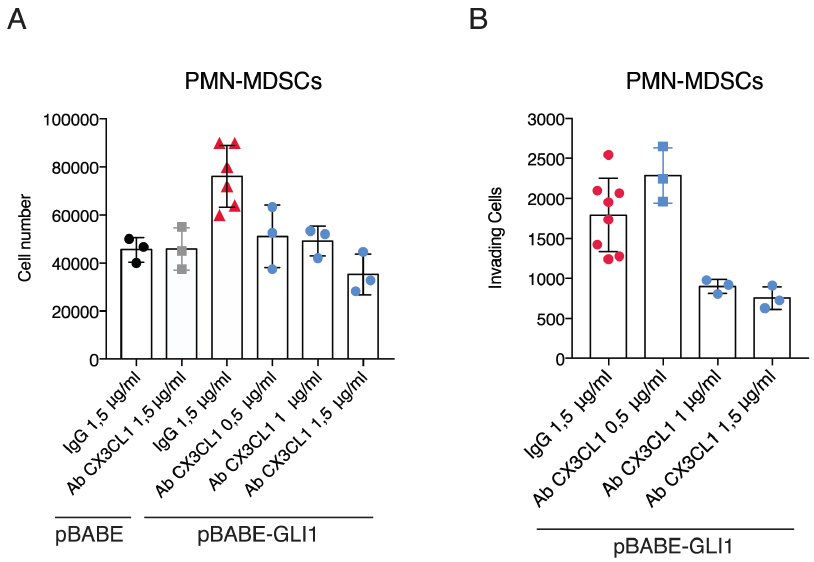


**Suppl. Figure 9. Titration of CX3CL1 neutralizing antibody in PMN-MDSC proliferation and invasion assays. A,** Cell number of PMN-MDSCs conditioned with media from B16F10 cells transduced with pBABE or pBABE-GLI1 and treated with increasing concentration of neutralizing CX3CL1 antibody or IgG isotype matched control. **B,** Invasion assay of PMN-MDSCs conditioned with media from B16F10 cells transduced with pBABE or pBABE-GLI1 and treated with increasing concentration of neutralizing CX3CL1 antibody (0.5, 1, 1.5 μg/ml) or IgG isotype matched control (1.5 μg/ml).

**
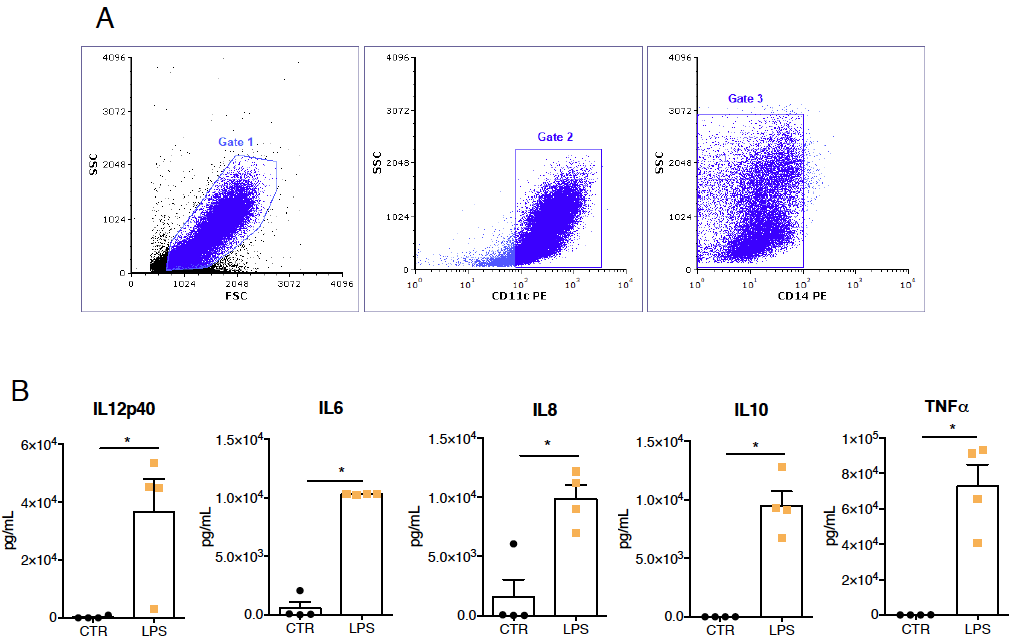
**

**Suppl. Figure 10. Differentiation of human moDCs. A,** Flow cytometry gating strategy used to evaluate the percentage of differentiated (CD11c+ CD14-) human monocyte-derived DCs after 7 days of incubation of CD14+ monocytes with GM-CSF and IL-4. DCs were included in Gate 1 (left dot plot), on the total of captured events, in a SSC (side scatter) versus FSC (forward scatter) plot. Gate 2 (middle dot plot) and Gate 3 (right dot plot) report the percentage of differentiated CD11c+ and CD14- DCs versus SSC, respectively. **B,** Concentration (pg/mL) of cytokines IL-12p40, IL-6, IL-8, IL-10, and TNFα, measured by Luminex assay in supernatants of unstimulated (CTR) or lipopolysaccharide (LPS)-activated moDCs. Mean ± SEM of data from 4 donors/condition is shown. Two-sample Mann-Whitney test was used. **p* < 0.05.


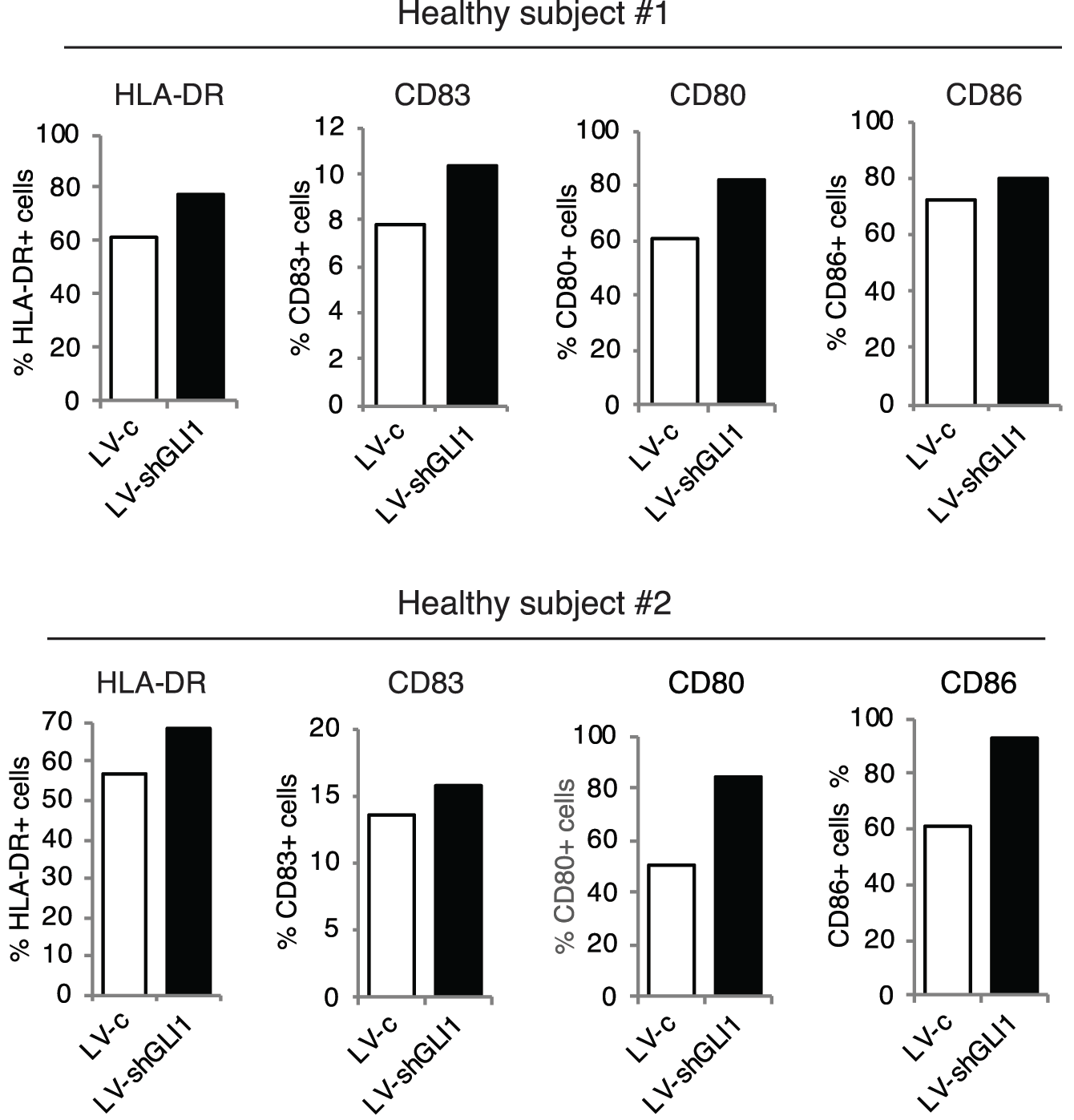


**Suppl. Figure 11. Immunophenotype of human monocyte-derived DCs (moDCs).** Percentage of cells positive for HLA-DR, CD83, CD80 and CD86. moDCs from two representative healthy subjects were cultured with conditioned media from SSM2c cells transduced with LV-c or LV-shGLI1.

**
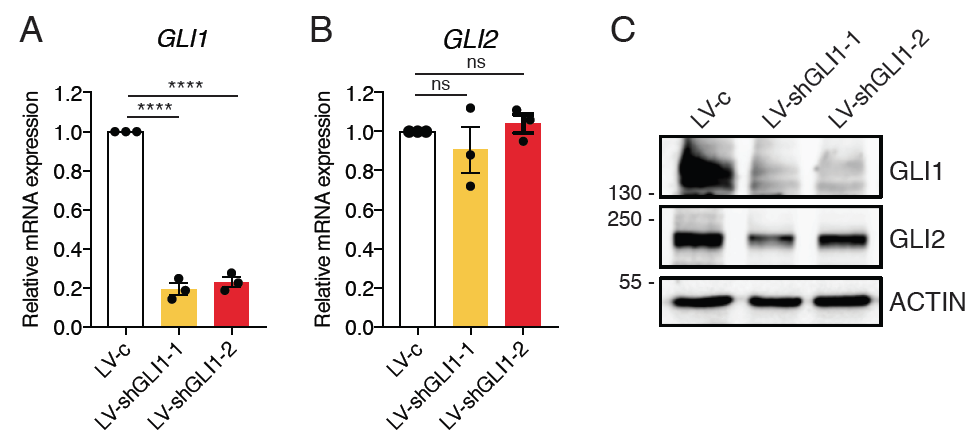
**

**Suppl. Figure 12. GLI2 does not compensate for the loss of GLI1. A-B**, Quantitative real-time PCR (qPCR) of *GLI1* (**A**) and *GLI2* (**B**) mRNA in human SSM2c melanoma cells transduced with LV-c, LV-shGLI1-1 or LV-shGLI1-2. Data represent mean ± SEM. *****p* < 0.0001; ns, not significant (one-way ANOVA). **C**, Western blot analysis of GLI1 and GLI2 in SSM2c melanoma cells transduced with LV-c, LV-shGLI1-1 or LV-shGLI1-2. ACTIN was used as loading control.

**
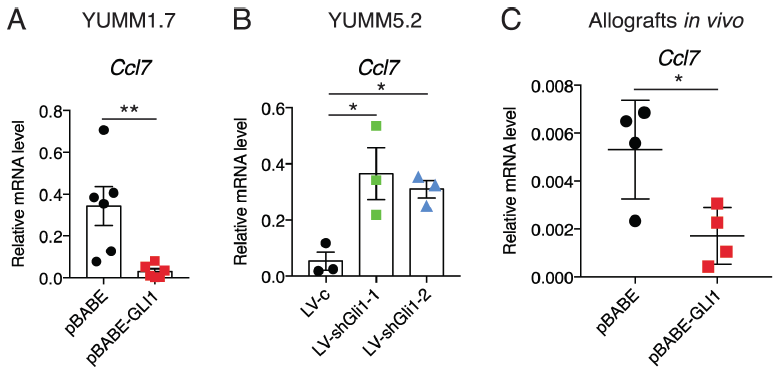
**

**Suppl. Figure 13. GLI1 negatively modulates the expression of *Ccl7* in melanoma cells and in allografts. A-B,** qPCR of *Ccl7* in murine melanoma cells YUMM1.7 (**A**) and YUMM5.2 (**B**) transduced as indicated. Data represent mean ± SEM. **C,** Quantitative real-time PCR (qPCR) of *Ccl7* in murine allografts injected with B16F10 cell transduced with pBABE or pBABE-GLI1. **p* < 0.05; ***p* < 0.01. Unpaired Student’s *t* test (**A,C**); one-way ANOVA (**B**).

**
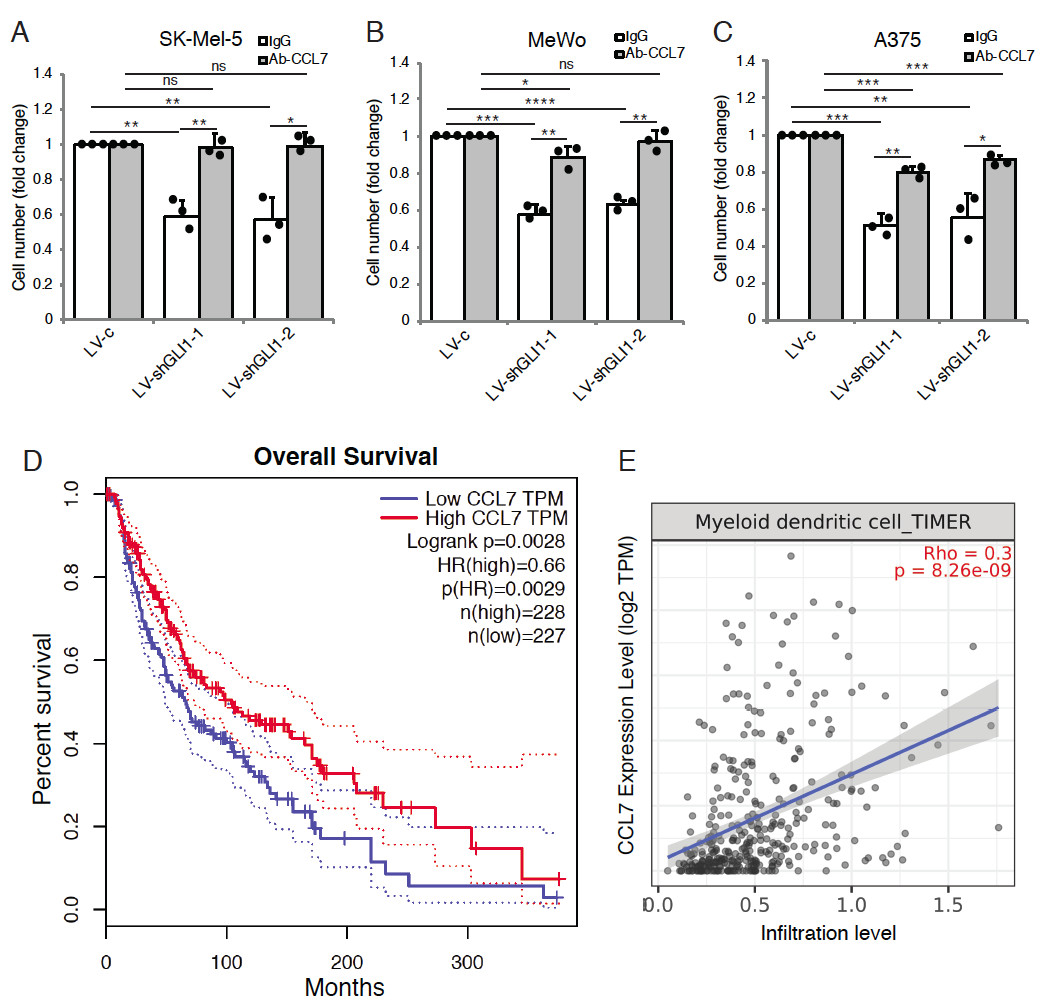
**

**Suppl. Figure 14. Effect of CCL7 in human melanoma. A-C,** Proliferation of SK-Mel-5 (**A**), MeWo (**B**) and A375 (**C**) cells cultured with conditioned media (CM) from SSM2c cells transduced with LV-c, LV-shGLI1-1 or LV-shGLI1-2 and treated with 10μg/ml CCL7 neutralizing antibody or IgG isotype matched control for 48 h. Cells cultured with LV-c CM were equated to 1. Note that CCL7 blocking antibody rescues the anti-proliferative effect of conditioned media from GLI1-silenced melanoma cells. **p* < 0.05; ***p* < 0.01; ****p* < 0.001; *****p* < 0.0001 (one-way ANOVA). **D**, Kaplan–Meier overall survival in melanoma patients with high (red, n=228) and low (blue, n=227) CCL7 expression (Logrank test p=0.0028) (dataset from cBioportal, https://www.cbioportal.org). **E**, Correlation between *CCL7* mRNA expression and dendritic cell infiltration in metastatic melanomas (n=368, p=8.26e^-9^) (dataset from TIMER 2.0, http://timer.cistrome.org).

**
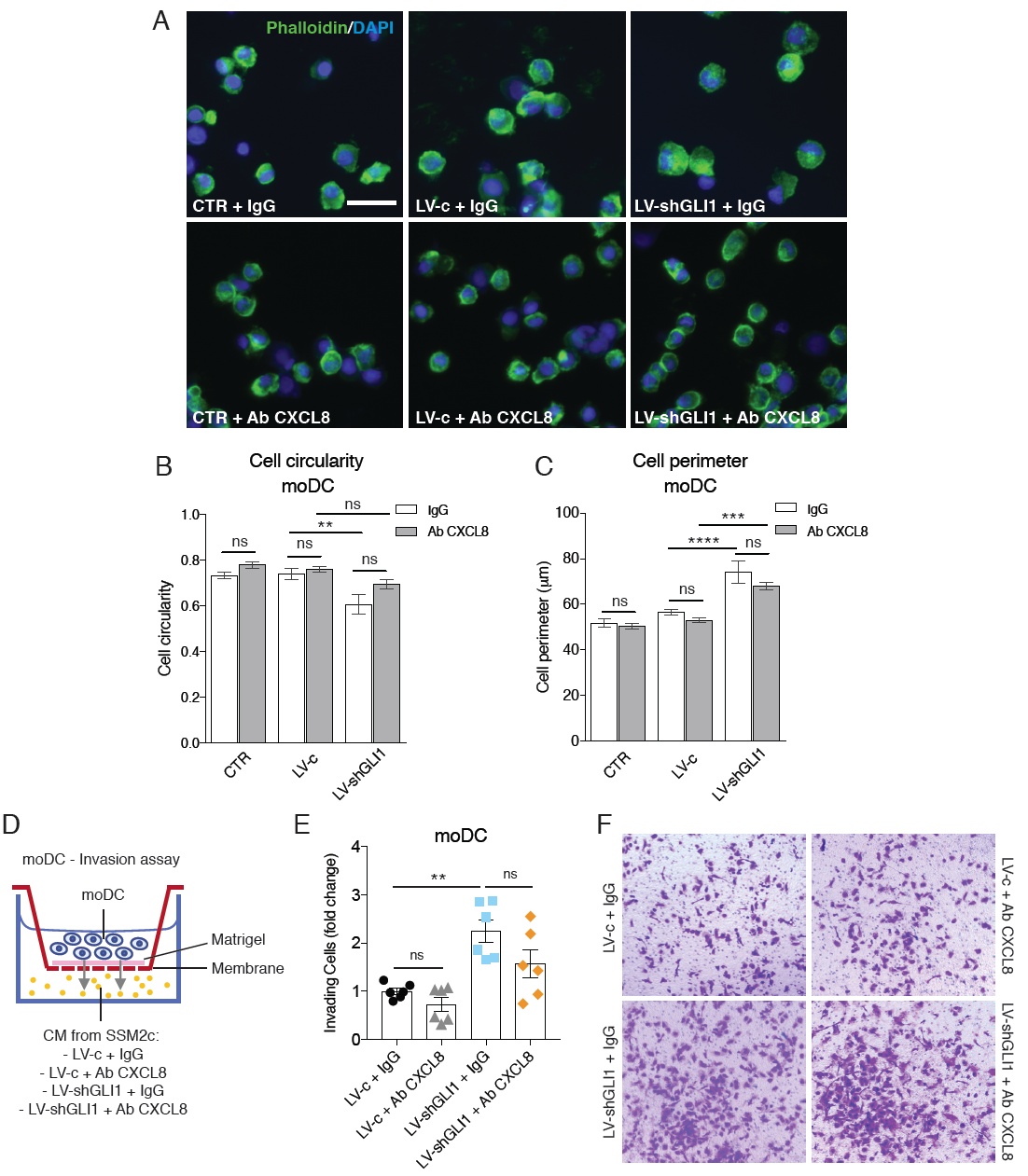
**

**Suppl. Figure 15. Effect of CXCL8 blockade on the activation of moDCs promoted by GLI1 silencing in melanoma cells. A,** Representative immunofluorescence images acquired at 20X magnification of moDCs cultured 48 h in RPMI media (CTR), CM from SSM2c melanoma cells transduced with LV-c or LV-shGLI1 and treated with neutralizing CXCL8 antibody or IgG isotype matched control. moDCs were stained with Phalloidin and counterstained with DAPI. Scale bar = 20 μm. **B,C** Cell circularity (4π*area/perimeter^2^) (**B**) and cell perimeter (μm) (**C**) analyses of DCs treated as indicated in (**A**) and quantified by ImageJ software. An average of 20 cells from 10-15 random glass slide areas was analyzed. Mean ± SEM from two donors of three independent experiments are reported. ***p* < 0.01; ****p* < 0.001; *****p* < 0.0001; ns, not significant (one-way ANOVA). **D,** Schematic representation of human DC invasion assay. Cells were seeded in the upper chamber and CM from SSM2c cells transduced with LV-c or LV-shGLI1 and treated with blocking CXCL8 antibody or IgG isotype in the lower chamber. Number of invading cells was counted after 48 h. **E,** Invasion assay of moDCs recruited after 48 h by CM from SSM2c cells transduced with LV-c or LV-shGLI1 and treated with blocking CXCL8 antibody or IgG isotype. Data represents mean ± SEM of at least three independent experiments. ***p* < 0.01; ns, not significant (one-way ANOVA). **F,** Representative pictures of (**E**). DAPI = 4’, 6-diamidino-2-phenylindole.


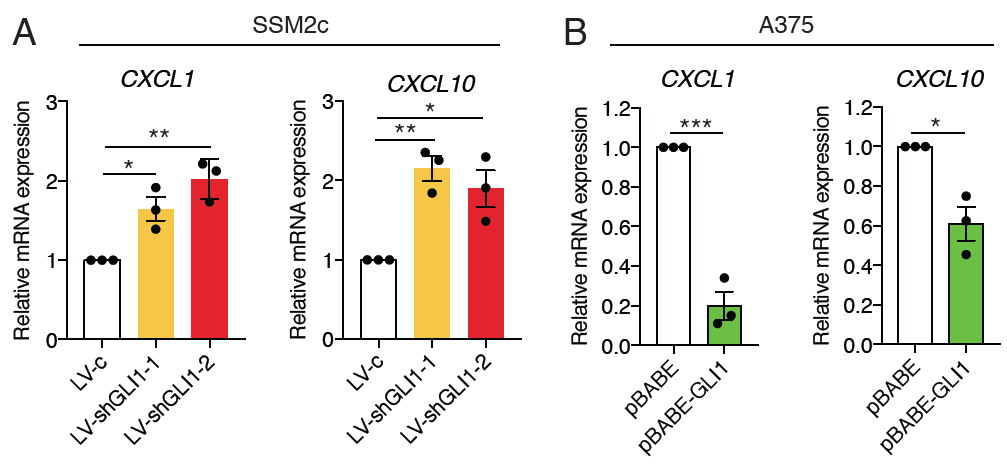


**Suppl. Figure 16. GLI1 negatively modulates the expression of *CXCL1* and *CXCL10*. A,** qPCR of *CXCL1* and *CXCL10* in patient-derived SSM2c human melanoma cells transduced with LV-c, LV-shGLI1-1 or LV-shGLI1-2 (referred to Fig. 7C). **B,** qPCR of *CXCL1* and *CXCL10* in A375 human melanoma cells transduced with pBABE or pBABE-GLI1 (referred to Fig. 7C). Data are expressed as fold change relative to control LV-c or pBABE, which were equated to 1. Gene expression is expressed as mean ± SEM. **p* < 0.01; ***p* < 0.01; ****p* < 0.001. One-way ANOVA in (**A**) and unpaired Student *t* test (**B**).

**SUPPLEMENTAL TABLES**

**Suppl. Table 1. List of primers used for qPCR.**

| **Gene** | **Primer Sequence (5’ to 3’)** |
| --- | --- |
| *mGli1* | FW: GCAACCTTCTTGCTCACACA |
|  | RV: GAAGGAATTCGTGTGCCATT |
| *mCx3cl1* | FW: CAGTGGCTTTGCTCATCCGCTA |
|  | RV: AGCCTGGTGATCCAGATGCTTC |
| *mCcl7* | FW: AAGATCCCCAAGAGGAATCTCAAG |
|  | RV: CAGACTTCCATGCCCTTCTTTG |
| *mGapdh* | FW: TGACCACAGTCCATGCCATC |
|  | RV: GACGGACACATTGGGGGTAG |
| *mActin* | FW: GGCTCCTAGCACCATGAAG |
|  | RV: GAAAGGGTGTAAAACGCAGC |
| *hGLI1* | FW: CCCAGTACATGCTGGTGGTT |
|  | RV: GCTTTACTGCAGCCCTCGT |
| *hGLI2* | FW: CTCAGCCCCGCTGATGTGGC |
|  | RV: TCAGCAGGTCCCCGTAGGGC |
| *hCX3CL1* | FW: CCACCTTCTGCCATCTGACT |
|  | RV: TCTCCAAGATGATTGCGCGT |
| *hCCL7* | FW: TTGCTCAGCCAGTTGGGATTA |
|  | RV: AGTCCTGGACCCACTTCTGT |
| *hCCL2* | FW: CCCAAAGAAGCTGTGATCTTCA |
|  | RV: TCTGGGGAAAGCTAGGGGAA |
| *hCCL20* | FW: GGCGAATCAGAAGCAGCAAGC |
|  | RV: ATTTGCGCACACAGACAACTTT |
| *hCXCL8* | FW: ACTCCAAACCTTTCCACCCC |
|  | RV: TTCTCAGCCCTCTTCAAAAACT |
| *hCXCL1* | FW: AGCTTGCCTCAATCCTGCATCC |
|  | RV: TCCTTCAGGAACAGCCACCAGT |
| *hCXCL10* | FW: GCAGTTAGCAAGGAAAGGTCTAAA |
|  | RV: CCATGTAGGGAAGTGATGGGAG |
| *hGAPDH* | FW: GACGCTGGGGCTGGCATTG |
|  | RV: GCTGGTGGTCCAGGGGTC |
| *hTBP* | FW: CAACAGCCTGCCACCTTAC |
|  | RV: CTGAATAGGCTGTGGGGTC |

**Suppl. Table 2. List of primary antibodies used for Western blotting.**

| **Protein/Antibody** | **Origin** | **Catalog number** | **Company** | **Identifier** |
| --- | --- | --- | --- | --- |
| GLI1 | Mouse | 2643 | Cell Signaling Technology | AB_2294746 |
| GLI2 | Goat | AF3635 | R&D Systems | AB_2111902 |
| ACTIN | Mouse | sc-47778 | Santa Cruz Biotechnology | AB_626632 |
